# Retinol binding protein 4 enhances cellular cholesterol uptake to facilitate influenza A virus infection

**DOI:** 10.1371/journal.ppat.1013623

**Published:** 2025-10-27

**Authors:** Hejiao Zhao, Yalu Zhang, Qingbing Han, Huanan Li, Wenjun Liu, Lei Sun, Yingli Shang

**Affiliations:** 1 Department of Preventive Veterinary Medicine, College of Veterinary Medicine, Shandong Agricultural University, Taian, Shandong, China; 2 Shandong Provincial Key Laboratory of Zoonoses, Shandong Agricultural University, Taian, Shandong, China; 3 CAS Key Laboratory of Pathogenic Microbiology and Immunology, Institute of Microbiology, Chinese Academy of Sciences, Beijing, China; 4 Savaid Medical School, University of Chinese Academy of Sciences, Beijing, China; 5 Shenzhen Medical Academy of Research and Translation (SMART), Shenzhen, Guangdong, China; 6 Shandong Public Health Clinical Center, Jinan, Shandong, China; 7 Institute of Immunology, Shandong Agricultural University, Taian, Shandong, China; University of Zurich: Universitat Zurich, SWITZERLAND

## Abstract

Viruses hijack host cell machinery to facilitate their own replication. Therefore, identifying key cellular factors and processes involved in viral infection is crucial for developing host-directed therapies. Herein, we demonstrate that retinol-binding protein 4 (RBP4), a lipocalin family member and major retinol carrier, is significantly induced by influenza A virus (IAV) infection in both cellular models and clinical patients. Moreover, RBP4 deficiency impairs IAV replication both *in vitro* and *in vivo*. Mechanistically, RBP4 promotes the expression of CD36, a cholesterol uptake receptor protein, thereby increasing cellular cholesterol levels. This elevation in cholesterol subsequently boosts cell-surface sialic acid levels, facilitating IAV attachment. Consequently, enforced expression of CD36 restores IAV replication in RBP4-deficient cells and mice. In summary, our study identifies RBP4 as a pivotal host factor that facilitates IAV infection by modulating cellular cholesterol homeostasis.

## Author summary

Cholesterol metabolism is critical in a variety of viral infections, and influenza A viruses (IAV) employ multiple strategies to hijack this metabolic pathway to evade host immunity. Here, we provide compelling evidence that retinol-binding protein 4 (RBP4) promotes the upregulation of CD36 expression, thereby enhancing cholesterol uptake and sialic acid receptor expression. This process facilitates IAV infection and replication. Our findings reveal a mechanism by which IAV exploits host factors to maintain its own replication and suggest that RBP4 may represent a promising target for the development of novel anti-IAV therapies.

## Introduction

The influenza virus, which causes influenza, affects nearly 10% of the world’s population annually and results in approximately half a million deaths each year and thus poses a significant threat to public health [1]. Immunocompromised individuals such as the elderly (>65 years old) and young children (< 5 years) are particularly prone to severe influenza virus-induced acute respiratory disease [2]. In addition, influenza virus is high susceptibility to mutation and reassortment, providing evolutionary flexibility that allows it to recombine different genotypes [3]. Some of these genotypes may be more pathogenic. For instance, certain influenza strains, such as the H1N1 strain or the related H7N9 and H5N1 strains, can cause pandemic infections that significantly impact a large proportion of the global population [4].

The influenza virus infects the host by attaching to the host cell and penetrating the membrane. This process begins when the viral hemagglutinin (HA) protein binds to sialic acid receptors on the cell surface, with human and animal influenza viruses exhibiting distinct receptor specificities [5]. Influenza A virus (IAV) proteins often interact with various host factors across different cellular compartments, subverting multiple cellular processes. This enables the virus to hijack the host cellular machinery and evade the host immune response. Additionally, both obesity and metabolic syndrome have been identified as independent risk factors for severe influenza virus infection [6,7]. For example, seasonal influenza affected 19% more obese people than those of normal weight, and obesity increased both the incidence of seasonal influenza and the mortality rate from respiratory illness [8]. Moreover, obese patients with influenza experience a longer viral shedding time compared to non-obese individuals [9]. Notably, recent study indicates that the cellular protein apolipoprotein E (APOE), a lipoprotein transport protein crucial for regulating cellular cholesterol metabolism, inhibits influenza virus infection and replication [10], suggesting that cholesterol homeostasis plays a role in influenza virus infection. Although aberrant lipid metabolism has been linked to various virus infections [11], the specific mechanisms by which host lipid metabolism influences influenza virus infection are still largely unclear.

Retinol-binding protein 4 (RBP4) belongs to the lipocalin family and serves as the major carrier protein for retinol in serum. It is predominantly secreted by hepatocytes and adipocytes [12,13]. RBP4 is responsible for transporting retinol from the liver to various tissues and facilitating its uptake into target cells via the receptor STRA6 [14,15]. Consequently, RBP4 levels are elevated in serum and adipose tissue in cases of obesity-induced insulin resistance and are linked to metabolic syndromes [16]. Additionally, macrophages are also significant sites of RBP4 expression [17]. Studies have shown that RBP4 can induce inflammatory responses in macrophages or endothelial cells through activation of toll-like receptors (TLRs) [18]. Thus, the functions of RBP4 can be either dependent on its role in retinoid homeostasis or independent of retinol transport. Furthermore, RBP4 expression is also correlated with infection by certain viruses [19–22]. For example, RBP4 levels increase during hepatitis C virus (HCV) infection, and its knockdown promotes HCV replication. Our recent research indicates that RBP4 suppresses porcine circovirus 2 (PCV2) infections *in vitro* and *in vivo* by regulating host autophagy processes [23]. Notably, RBP4 levels are significantly increased in hospitalized patients diagnosed with influenza A (H1N1) pdm09 virus infection, correlating with clinical severity of the infection [24]. However, the impact of RBP4 on influenza virus infection and replication is yet to be understood.

In this study, we found that RBP4 levels are significantly increased in response to IAV infection. Overexpression of RBP4 enhances IAV infection while deficiency of RBP4 reduces IAV infection *in vivo* and *in vitro*. RBP4 plays a role in the early steps of IAV lifecycle, particularly in enhancing the viral attachment to the cell membrane. Mechanistically, RBP4 upregulates the expression of CD36, a receptor protein involved in cholesterol uptake, which in turn increases cellular cholesterol levels. This rise in cholesterol levels leads to increases in cell-surface sialic acids, the influenza virus receptors, thereby promoting influenza virus binding and replication. Overall, our findings identify RBP4 as a critical host factor that regulates influenza virus infection and uncover a previously unrecognized function of RBP4 in facilitating virus infection through the modulation of cholesterol metabolism.

## Results

### RBP4 acts as a host factor to facilitate IAV infection

**Fig 1:**
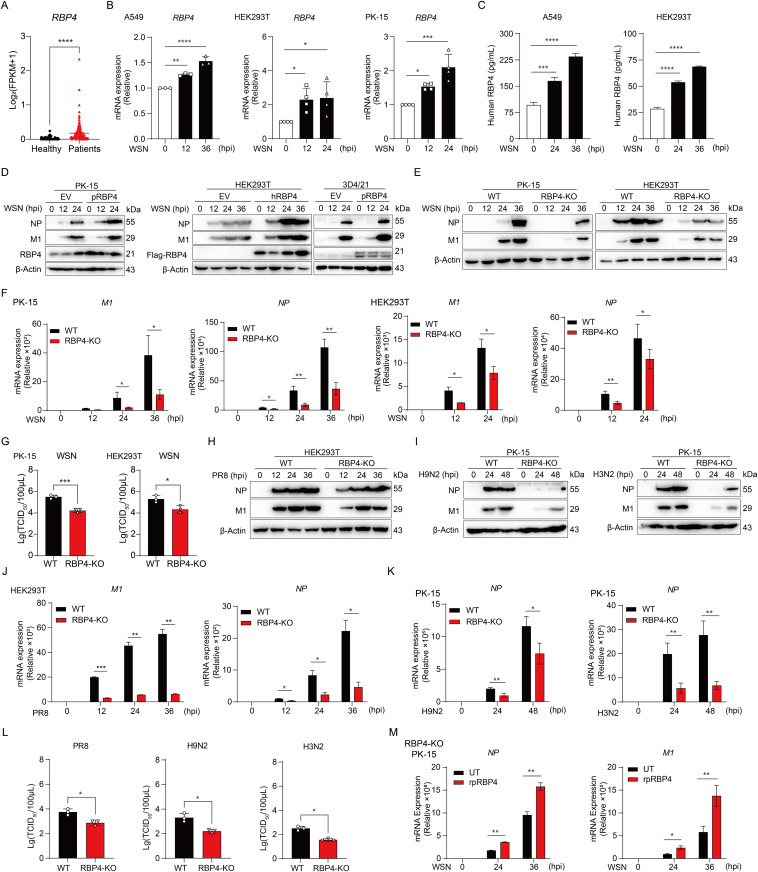
RBP4 promotes IAV replication *in vitro.* (A) Statistical analysis of the FPKM values of RBP4 in the blood of patients with influenza infection (n = 148) and healthy people (n = 82) obtained from four online GEO databases (GSE155635, GSE158592, GSE196350 and GSE213168). (B) qPCR analysis of RBP4 mRNA levels in A549, HEK293T and PK-15 cells upon WSN (MOI = 0.1) infection at indicated periods. (C) ELISA of human RBP4 in supernatants of A549 and HEK293T cells infected with WSN (MOI = 0.1) for indicated periods. (D) Immunoblotting analysis of NP and M1 protein levels in HEK293T, PK-15 and 3D4/21 cells transfected with RBP4 or control plasmids for 12 hours, following infection with IAV WSN strain (MOI = 0.1) for various periods. (E, F) Immunoblotting (E) or qPCR analysis (F) of NP and M1 protein levels or mRNA expression in WT and RBP4-defecient PK-15 cells or HEK293T cells infected with IAV WSN strain (MOI = 0.1) for indicated periods. (G) Plaque assay of viral titers in WT and RBP4-deficient HEK293T and PK-15 cells infected with WSN (MOI = 0.1) for 24 hours. (H) Immunoblotting analysis of NP and M1 protein expression in WT and RBP4-deficient HEK293T cells infected with IAV PR8 strain (MOI = 0.1) for indicated periods. (I) Immunoblotting analysis of WT and RBP4-deficient PK-15 cells infected with IAV H3N2 (MOI = 0.1) or H9N2 (MOI = 0.1) for indicated periods. (J-L) RT-qPCR analysis of NP and M1 mRNA expression (J) or plaque assay of virus titers (L, left) in WT and RBP4-deficient HEK293T cells infected with IAV PR8 strain (MOI = 0.1) (J, L, left) or in WT and RBP4-deficient PK-15 cells infected with IAV H9N2 strain (K, left and L, middle) or H3N2 strain (K, right and L, right). (M) RT-qPCR analysis of mRNA levels of NP and M1 in RBP4-deficient PK-15 (RBP4-KO PK-15) cells infected with WSN (MOI = 0.1) for indicated periods. After 6 hours of WSN infection, cells were treated with or without recombinant porcine RBP4 (rpRBP4). Data are representative of two independent experiments (D, E, H, I) or pooled from at least three independent experiments (B, C, F, G, J-M, mean ± SD). *p* values were calculated using One-way ANOVA (B and C) or unpaired student’s *t*-test (A, F, G, J-M). *p < 0.05, **p < 0.01, ***p < 0.001, ****p < 0.0001.

The increase of RBP4 has been implicated in patient diagnosed with influenza A virus (IAV) infection [24]. To validate the correlation between RBP4 and influenza virus infection, we collected the clinical RNA sequencing (RNA-Seq) data of blood samples from healthy people and patients infected with influenza virus from 2018 to 2022 from the GEO database. We then performed statistical analysis of RBP4 expression. The results showed that RBP4 expression was significantly higher in the blood of IAV-patients than in healthy individuals (Fig 1A), which is consistent with previous findings in nasopharyngeal aspirate samples from influenza patients [24]. Next, we examined the mRNA and protein levels of RBP4 in different type of cells, including human A549 cells, human HEK293T cells and porcine PK-15 cells, infected with influenza virus. We found that RBP4 expression was markedly induced at both the mRNA and protein levels in response to IAV infection in multiple cell types (Fig 1B and 1C). These results suggest that influenza virus infection promotes RBP4 expression both *in vitro* and *in vivo*. To elucidate the mechanisms underlying IAV-mediated RBP4 induction, we first asked whether the increase of RBP4 requires active virus replication or can be elicited solely by incoming virions. qPCR analysis in HEK293T cells infected with WSN showed significant RBP4 induction as early as 6 hours post-infection (S1A Fig), implying that virus replication is likely dispensable for this response. Bioinformatic analysis of the human *RBP4* promoter identified multiple putative binding sites for key transcriptional factors including NF-κB, AP-1, C/EBPα and PPARα (S1B Fig). We therefore examined whether the these signaling pathways participate in regulation of RBP4 transcription. Immunoblotting analysis revealed time-dependent phosphorylation of p65, p38, ERK and mTOR upon IAV infection (S1C Fig). Consistently, pharmacological inhibition of NF-κB, MAPKs and mTOR signaling markedly attenuated RBP4 induction in A549 cells (S1D Fig). Collectively, these data indicate that IAV likely engages multiple signaling cascades to regulate RBP4 expression at transcriptional level.

To further ascertain the role of RBP4 as a pivotal host factor in IAV infection, we initially overexpressed RBP4 in multiple cell lines, including A549, HEK293T, PK-15 and 3D4/21 cells, and were subsequently infected with H1N1 IAV (strain WSN). Immunoblotting analysis showed that IAV replication was significantly elevated in cells overexpressing RBP4 relative to control cells (Figs 1D and S2A), suggesting that RBP4 may promotes influenza virus replication *in vitro*. To further validate these findings, we generated RBP4 knockout (RBP4 KO) cell lines using CRISPR-Cas9 technology targeted specifically at RBP4 [23] and verified the deficiency of RBP4 by immunoblotting and DNA sequencing (S2B and S2C Fig). Both RBP4 KO and wild-type (WT) cell clones were infected with WSN and then the cell lysates were analyzed by immunoblotting to assess the viral protein expression. Our results indicated a reduced expression of M1 and NP proteins in RBP4 KO cells compared with WT cells (Fig 1E). Furthermore, qPCR and plaque assays showed that the NP and M1 gene copy numbers, together with virus titers, were markedly lower in RBP4 KO cells than in WT controls (Fig 1F and 1G). The same trend was observed in RBP4 knockdown A549 cells (S2D and S2E Fig). Collectively, the overexpression, knockdown and knockout data demonstrate that RBP4 facilitates influenza virus infection.

To further determine whether the role of RBP4 is conserved across different IAV subtypes, we infected both WT and RBP4 KO cell lines with the human influenza H1N1 strain PR8, as well as swine-origin H3N2 and avian-origin H9N2 strains. qPCR, plaque assays and immunoblotting revealed that RBP4 deletion substantially decreased viral RNA levels, infectious titers, and viral-protein expression across all tested strains relative to wild-type cells (Fig 1H–1L), indicating that the pro-viral effect of RBP4 is not subtype-restricted but broadly conserved. Moreover, exogenous recombinant RBP4 restored viral replication in RBP4 KO cells (Fig 1M). Taken together, these data established that RBP4 acts as a host dependency factor that promotes replication of diverse IAV strains *in vitro*.

### RBP4 deficiency diminished IAV infection and alleviated disease pathology

**Fig 2:**
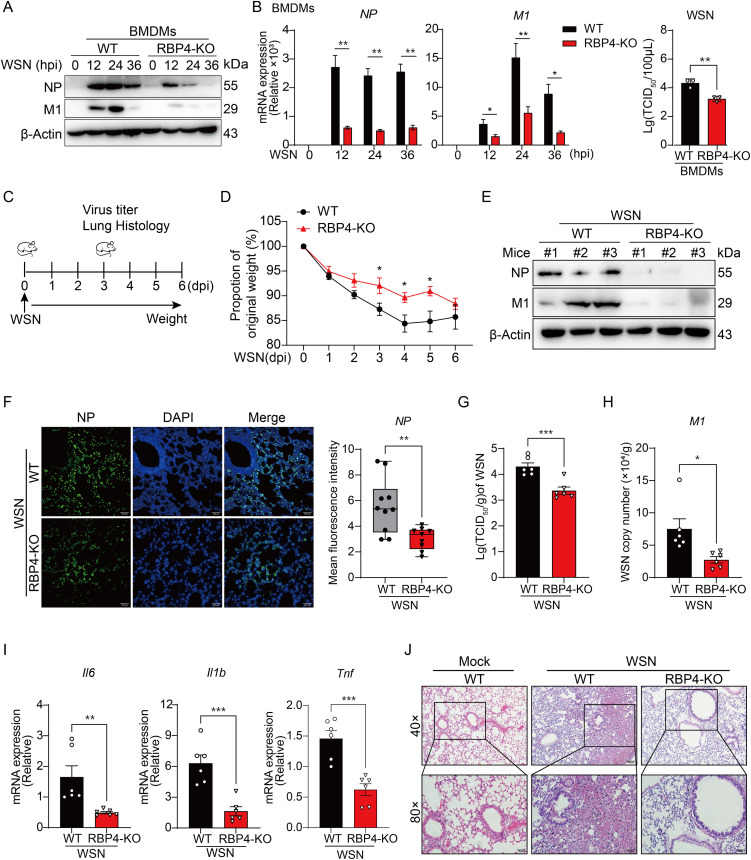
RBP4 deficiency attenuates IAV replication *in vivo.* (A, B) Analysis of NP and M1 protein levels and mRNA expression or plaque assay of virus titers in BMDMs derived from WT and RBP4-deficient mice infected with IAV WSN strain (MOI = 0.5) at indicated time points. Representative immunoblotting results from two independent experiments (A) and pooled qPCR data and virus titers (B) from three independent experiments (mean ± SD) were shown, respectively. (C) Schematic diagram of the influenza virus infection model in mice. WT and RBP4-deficient mice were intranasally infected with 1 × 10^4^ PFU of WSN. Lungs were collected 3 days post-infection, and body weight was monitored daily for 6 days. (D) Body weight changes in mice infected with IAV WSN as described in (C) (mean ± SEM, n = 6). (E) Immunoblotting analysis of NP and M1 protein expression in lungs of mice infected with IAV as in C (n = 3). (F) Immunofluorescence of NP proteins in lung tissues from WT and RBP4-deficient mice infected IAV as described in (C). Scale bars, 100 μm. Mean fluorescence intensity was calculated from two independent experiments (right). (G-J) Plaque assay analysis of virus titers (G), qPCR analysis of M1 copy number (H) and inflammatory cytokine expression (I) in lung tissues or histopathological analysis of lung tissues from WT and RBP4-deficient mice infected with WSN at day 3 post-infection. Each dot represents an individual mouse. Scale bars, 100 μm in J. Data are representative of two independent experiments (E-J, mean ± SEM). *p < 0.05, **p < 0.01, ***p < 0.001 (Student’s *t*-test).

Having established that RBP4 enhances influenza virus infection *in vitro*, we next aimed to investigate its role *in vivo*. Firstly, we isolated bone marrow-derived macrophages (BMDMs) from both WT and RBP4-deficient mice and confirmed the absence of RBP4 by immunoblotting (S2F Fig). Upon WSN challenge, RBP4-null BMDMs exhibited markedly lower M1 and NP protein levels, accompanied by reduced IAV mRNA abundance and viral titers (Fig 2A and 2B). These data suggest that RBP4 also promotes IAV replication in murine primary macrophages. We then intranasally infected WT and RBP4-deficient mice with WSN for 3 days to test virus replication, monitored disease progression, and recorded body weight changes for 6 days of infection (Fig 2C). Notably, WT mice exhibited more severe weight loss following WSN infection compared to RBP4-deficient mice (Fig 2D), implying that RBP4 deficiency mitigates disease induced by IAV. We also collected lungs infected with WSN for 3 days to grinding and lysis. Immunoblotting analysis revealed a significantly lower expression of NP and M1 of IAV in the lungs of RBP4-deficient mice (Fig 2E). Similarly, immunofluorescence staining for the viral protein NP indicated reduced viral antigen levels in lungs of RBP4-deficient mice (Fig 2F). These data demonstrate that RBP4 facilitates influenza virus infection *in vivo*. Concurrently, IAV titers in the lungs were significantly diminished compared to those in WT mice (Fig 2G), and IAV mRNA levels were lower in the lungs of RBP4-deficient mice (Fig 2H). These findings strengthen the physiological relevance of RBP4 in IAV-infected regulation. In addition, the expression of pro-inflammatory cytokines, such as *Il1b*, *Tnf* and *Il6,* was also reduced in RBP4-deficient mice on day 3 post-infection, indicating a decrease inflammation (Fig 2I). We then observed pathological changes in the lungs, histopathological examination revealed that the lungs of RBP4-deficient mice displayed less damage and inflammation compared to those in WT mice (Fig 2J). As with H1N1 infection, H9N2-infected RBP4-deficient mice exhibited less replication than WT mice (S2G Fig). These data suggest that the absence of RBP4 significantly impaired the replication of influenza viruses in the lungs of mice. Altogether, these observations in IAV-infected RBP4-deficient mice further corroborate our conclusion that RBP4 plays a pivotal role in promoting IAV infection *in vivo.*

### RBP4 regulates the expression of viral receptors to influence viral attachment

**Fig 3:**
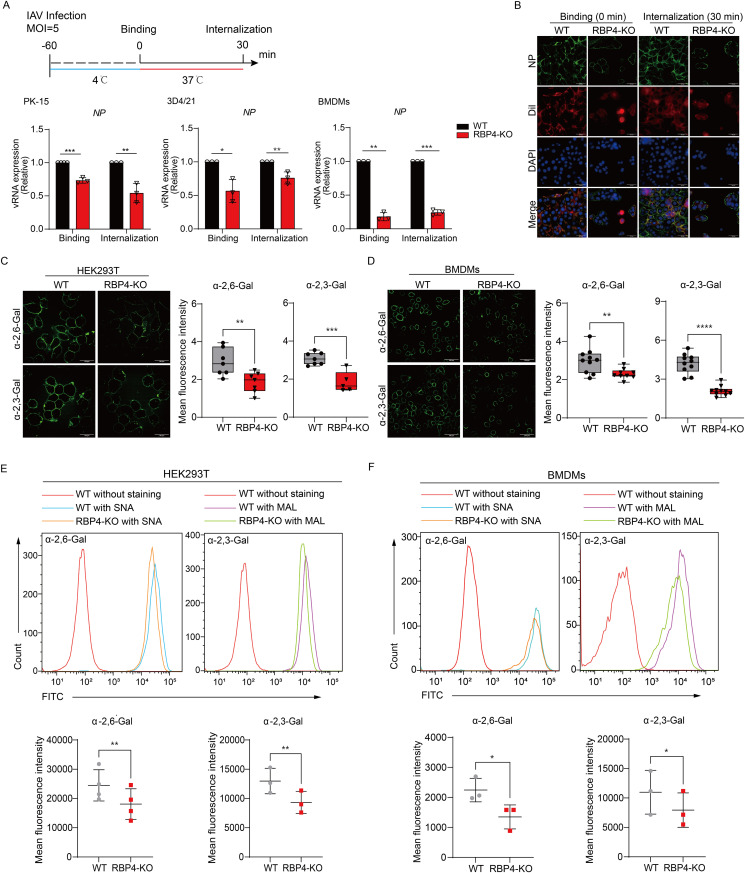
RBP4 enhances IAV attachment by upregulating sialic acid expression. (A) Schematic illustration of IAV attachment and internalization, along with qPCR analysis of viral RNA (vRNA) levels during these stages in various cell types. WT and RBP4-deficient cells (as indicated) were infected with IAV WSN strain (MOI = 5). Data are pooled from three independent experiments (mean ± SD). (B) Immunofluorescence staining of viral NP proteins (green) in WT and RBP4-deficient PK-15 cells infected with IAV as described in (A). Cell membranes were stained with Dil dye (red), and nuclei were counterstained with DAPI (blue). Scale bars, 100 μm. (C, D) Confocal microscopy images (C and D) of α-2,3-linked SA (stained with MAL) or α-2,6-linked SA (stained with SNA) in WT and RBP4-deficient HEK293T cells (C) or in WT and RBP4-deficient BMDMs (D). Scale bars, 100 μm. (E, F) Flow cytometry analysis (E and F) of α-2,3-linked SA or α-2,6-linked SA in the same cell types as in (C and D). Mean fluorescence intensity of α-2,3-linked or α-2,6-linked SA was quantified using Image J software (for confocal microscopy) or FlowJo software (for flow cytometry). Data are representative (B, C and D, left, E and F, upper) or pooled from at least three independent experiments (C and D, right, E and F, lower). *p < 0.05, **p < 0.01, ***p < 0.001, ****p < 0.0001 (Student’s *t*-test).

To determine the specific stage of the IAV replication impacted by RBP4, we performed a time-of-addition assay in HEK293T cells. Viral RNA (vRNA) and viral protein levels in cell lysates were then assessed by qPCR or immunoblotting analysis within 3 hours post IAV infection. We found a decreased mRNA and protein expression of M1 or NP in RBP4 KO cells (S2H and S2I Fig). Additionally, immunofluorescence staining for NP, predominantly localized to the nuclei during the early stage of infection, revealed that RBP4 deficiency significantly reduced the number of fluorescent nuclei (S2J Fig). These data suggest that RBP4 plays a key role in the early steps of IAV infection. It has been well known that the initial stages of influenza virus infection include attachment to sialic acid (SA) receptors on the cell surface, followed by rapidly endocytosis [25]. To investigate whether RBP4 influences viral attachment or internalization, we quantified the vRNA levels by qPCR in attachment and internalization assay in multiple cell types. We found decreased vRNA in RBP4-deficient cells compared to WT cells, suggesting that RBP4 deficiency impairs viral attachment (Fig 3A). Immunofluorescence analysis of NP protein during the early stage of IAV infection revealed significantly reduced fluorescence intensity on the surface of RBP4-deficient cells (Fig 3B), confirming that RBP4 expression contributes to influenza virus attachment. Moreover, exogenous recombinant RBP4 also enhanced IAV attachment in RBP4-deficient cells (S2K Fig). Collectively, these data suggest that RBP4 promotes the early steps of IAV lifecycle through modulating virus binding.

Given that the interaction between viral hemagglutinin (HA) and SA receptors on the cell surface is the initial step in invasion of host cells by IAV, we therefore investigated whether RBP4 affected the expression of these SA receptors. To do this, we used two specific lectins: Maackia amurensis lectin (MAL), which specially binds to α-2,3-SA, and Sambucus nigra lectin (SNA), which binds to α-2,6-SA, to assess the differential expression of surface SA on both WT and RBP4-deficient cells. Immunofluorescence analysis showed that the distribution of both α-2,3-linked SA and α-2,6-linked SA reduced on the surface of RBP4-deficient cells when compared to WT cells (Fig 3C and 3D). Quantitative flow cytometry analysis confirmed a significant reduction in the mean fluorescence intensity of surface SA of RBP4-deficient cells (Fig 3E and 3F). To test whether RBP4 regulates viral infection by additional cellular processes, we expanded our analysis to two genetically unrelated respiratory viruses with distinct entry requirements: Sendai virus (SeV), which strictly requires α-2,3-linked sialic acids [26], and vesicular stomatitis virus (VSV), which binds non-sialic acid receptors [27]. Although RBP4 depletion reduced surface α-2,3-sialic acid and prevented SeV attachment, it unexpectedly increased its replication and suppressed replication of the pH-dependent VSV (S2L and S2M Fig). These data indicate that RBP4 not only governs receptor availability, but also modulates later, sialic acid-independent stages of infection. Taken together, these results suggest that the restricted IAV infection observed in RBP4-deficient cells is likely due to alternations in the expression levels of cell surface SA receptors.

### RBP4 maintains cholesterol levels and enhances CD36 expression

**Fig 4:**
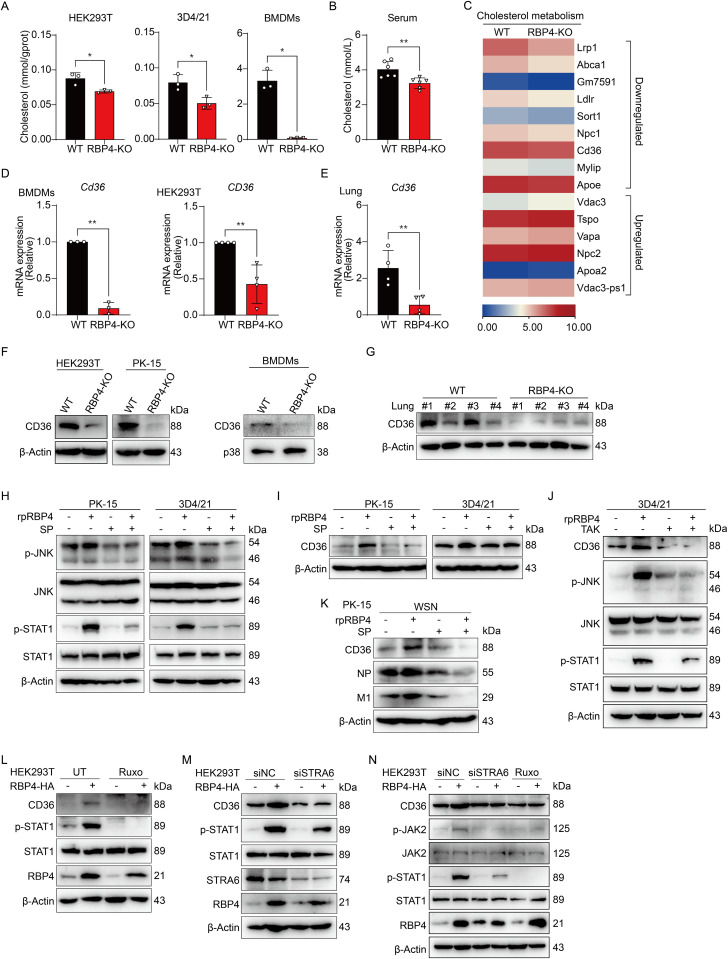
RBP4 improves CD36 expression and cholesterol level by activating STAT1. (A, B) Quantification of total cholesterol levels in multiple WT and RBP4-deficient cell types (A) and in serum from WT and RBP4-deficient mice (B). (C) Heatmap representation of RNA sequencing analysis showing differentially expressed genes related to cholesterol metabolism in WT and RBP4-deficient BMDMs. (D, E) qPCR analysis of CD36 mRNA expression in WT and RBP4-deficient BMDMs (D, left) and HEK293T cells (D, right), or in lung tissues from WT and RBP4-deficient mice (E). (F, G) Immunoblotting analysis of CD36 protein levels in multiple WT and RBP4-decficent cells as indicated (F) or in lung tissues from WT and RBP4-deficient mice (G). (H to J) Immunoblotting analysis of protein levels of CD36, total JNK, phosphorylated (p)-JNK, STAT1, and p-STAT1 as indicated in PK-15 or 3D4/21 cells pretreated with SP600125 (SP, 10 μM) or Resatorvid (TAK, 1 μM) for 2 hours, followed by treatment with rpRBP4 for 24 hours. (K) Immunoblotting analysis of protein expression of CD36, NP and M1 in PK-15 cells treated as described in (H), followed by infection with IAV WSN strain (MOI = 0.1) for 24 hours. (L) Immunoblotting analysis of CD36, p-STAT1, total STAT1 and RBP4 protein levels in HEK293T cells transfected with RBP4 expression plasmid or control empty vector, followed by treatment with Ruxolitinib (Ruxo, 5 μM) for 2 hours. (M) Immunoblotting analysis of CD36, p-STAT1, STAT1, STRA6 and RBP4 protein levels in HEK293T cells co-transfected with RBP4 expression plasmid and siRNA for STRA6 or negative control for 36 hours. (N) Immunoblotting analysis of CD36, p-JAK2, JAK2, p-STAT1, STAT1 and RBP4 protein levels in HEK293T cells transfected with RBP4 expression plasmid or control empty vector, followed by treatment with Ruxo (5 μM) for 2 hours or transfected with siRNA for STRA6 or negative control for 36 hours. Data are pooled from three independent experiments (A, B, D, E, mean ± SD) or representative of two independent experiments (F-N). *p < 0.05, **p < 0.01 (Student’s *t*-tes*t*).

Next, we aimed to determine how RBP4 affects SA receptor expression. Previous studies indicate that RBP4 levels are associated with dyslipidaemia and increased cholesterol levels [16]. Moreover, RBP4 has been implicated in lipid metabolism, contributing to metabolic disorders [28]. Since that cholesterol is an essential component of lipid rafts on the plasma membrane, which facilitate IAV binding and entry [29], we hypothesized that RBP4 might influence SA receptor expression and IAV binding by regulating cholesterol metabolism. To test this, we first measured the cellular cholesterol in WT and RBP4-deficiecnt cells. We found that cholesterol levels were lower in multiple RBP4-deficient cells compared to WT cells (Fig 4A), indicating that RBP4 is involved in maintaining cholesterol levels. This was further supported by *in vivo* data showing reduced serum cholesterol levels in RBP4-deficient mice (Fig 4B), confirming the role of RBP4 in cholesterol accumulation.

To dissect how RBP4 affects cholesterol levels, we conducted RNA sequencing (RNA-Seq) on WT and RBP4-deficient BMDMs. Global transcriptional profiling revealed a selective reshaping of genes involved in cholesterol metabolism in RBP4-deficient cells (Fig 4C). In particular, the scavenger receptor CD36 and the lipid transporter ApoE were markedly downregulated in RBP4-deficient BMDMs (Figs 4D, left and S3A), whereas the canonical cholesterol-synthesis enzymes HMGCR and DHCR24, together with the cholesterol uptake and efflux receptor SR-A, remained unperturbed (S3B Fig). These findings were further confirmed by qPCR and immunoblotting analysis both *in vitro* and *in vivo*, which revealed that RBP4 deficiency significantly reduced CD36 expression (Fig 4D–4G), a receptor that is crucial for cholesterol uptake. Together, these results demonstrate that RBP4 likely enhances cholesterol uptake by upregulating CD36 transcription.

As a secretory protein, RBP4 binds to its receptors TLR4/2 or STRA6 at the cell membrane to activate downstream signaling pathways, thereby regulating gene expression [30,31]. In fact, a previous study has shown that RBP4 can activate the JNK-STAT1 signaling pathway to promote CD36-mediated cholesterol uptake in macrophages [32]. To investigate whether STAT1 is involved in RBP4-induced CD36 upregulation, we treated PK-15 cells or 3D4/21 cells with recombinant porcine RBP4 protein (rpRBP4) and assessed CD36 expression. RBP4 treatment augmented STAT1 phosphorylation and increased CD36 expression, which were diminished by pretreatment of SP600125, a well-known chemical inhibitor of JNK (Figs 4H and 4I, S3C and S3D). This data suggests that RBP4 likely promotes the activation JNK-STAT1 axis to regulate CD36 expression in PK-15 cells and 3D4/21 cells. In macrophages, which lack STRA6, apo-RBP4 engages Toll-like receptors (TLR4) to trigger inflammatory responses [18]. To further determine whether TLR4 engagement is required for RBP4-mediated JNK/STAT activation and downstream CD36 induction, we treated 3D4/21 cells with TAK242, a selective antagonist of TLR4. TAK242 abolished RBP4-triggered phosphorylation of JNK and STAT1 and reversed CD36 up-regulation (Fig 4J), demonstrating that TLR4 signaling is indispensable for these events. These data suggest that RBP4 ignites the TLR4-JNK-STAT1 axis to drive CD36 expression in macrophages. To further determine the impact of RBP4-mediated induction of CD36 on IAV replication, we treated PK-15 cells with rpRBP4 protein and the JNK inhibitors before IAV infection. Immunoblotting analysis showed that RBP4 promoted viral replication, which was attenuated by treatment of the JNK inhibitors SP600125 (Fig 4K). In brief, these data reveal that RBP4 enhances CD36 expression through the activation of TLR4-JNK-STAT1 pathways, thereby contributing to IAV replication in macrophages.

Unlike macrophages, human HEK293T cells lack TLR receptors but express the STRA6 receptor at a relatively high level (S4A Fig). Given that RBP4 deficiency also reduces CD36 expression and STAT1 activation in HEK293T cells (Figs 4F and S4B), we therefore explored whether RBP4 activates STRA6 to regulate CD36 expression in these cells. Indeed, overexpression of RBP4 in HEK293T cells not only promotes CD36 expression but also activates STAT1 phosphorylation (Fig 4L). Consistently with this observation, RBP4 expression significantly enhances the CD36 promoter activity in HEK293T cells (S4C Fig). Interestingly, previous studies show that RBP4 can activate the JAK2-STAT signaling pathway via its STRA6 in pancreatic β-cell, HepG2 or in NH3T3 cells [33–36]. To test whether RBP4-induced CD36 expression in HEK293T cells requires JAK-STAT signaling, we treated the cells with ruxolitinib, a specific JAK inhibitor. Ruxolitinib treatment markedly attenuated RBP4-driven CD36 induction and concurrently blocked STAT1 phosphorylation (Fig 4L), confirming that the JAK-STAT1 axis is essential for RBP4-mediated CD36 induction in HEK293T cells. Consistently, STRA6 knockdown diminished RBP4-induced CD36 expression and suppressed both JAK2 and STAT1 activation (Fig 4M and 4N), supporting the notion that RBP4 binds STRA6 to activate the JAK2-STAT1 axis and modulate CD36 transcription in HEK293T cells. Collectively, these data demonstrate that RBP4 modulates CD36 transcription through binding to either TLR4 or STRA6 receptors, which subsequently activate the JNK-STAT1 or JAK2-STAT1 pathways in different cell types.

### RBP4 promotes CD36-mediated cholesterol uptake to regulate SA receptor expression

**Fig 5:**
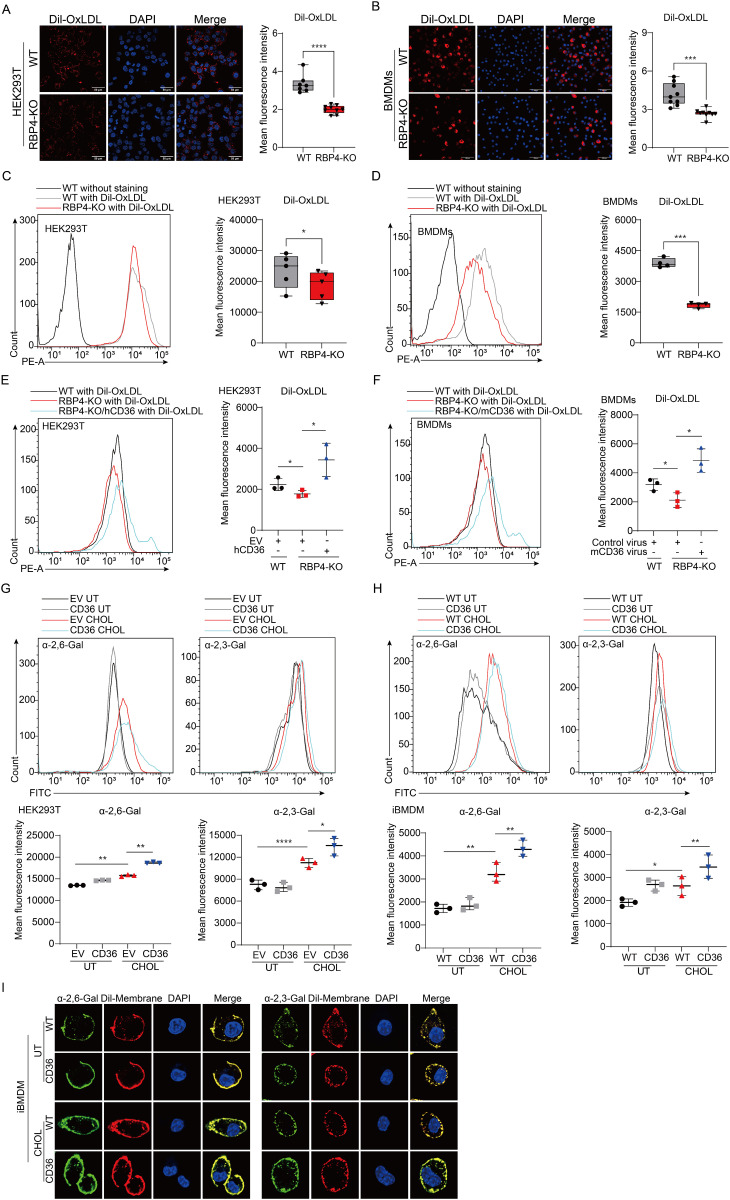
RBP4 enhances SA expression by promoting cholesterol uptake. (A to D) Confocal microscopy (A and B) or flow cytometry (C and D) analysis of cholesterol uptake by Dil-OxLDL in WT and RBP4-deficient HEK293T cells or BMDMs. Scale bars, 100 μm. Statistics of mean fluorescence intensity was calculated by Image J software (A and B, right) or Flow Jo software (C and D, right). (E, F) Flow cytometry analysis of cholesterol uptake by Dil-OxLDL in WT, RBP4-deficient and CD36 overexpressing RBP4-deficient HEK293T cells (E) and BMDMs (F). Mean fluorescence intensities were quantified with Flow Jo software. (G, H) Flow cytometry analysis of SA expression in HEK293T cells (G, upper) transfected with hCD36 plasmid or in stable CD36-overexpressing iBMDMs (H, upper). Cells were left untreated or incubated with cholesterol for 1 hour at 37°C, followed by staining with SNA or MAL. Mean fluorescence intensities were quantified by Flow Jo software (lower panels). (I) Confocal microscopy analysis of SA expression and distribution in WT and CD36-overexpressing iBMDMs. Cells were left untreated or incubated with cholesterol for 1 hour at 37°C, followed by staining with SNA or MAL (green). Cell membranes were stained with Dil dye (red), and nuclei were counterstained with DAPI (blue). Data are representative (A-F, left, G, H, upper and I) or pooled from at least three independent experiments (A-F, right, G and H, lower, mean ± SD). *p < 0.05, **p < 0.01, ***p < 0.001 (Student’s *t*-test).

Having known that RBP4 promotes CD36 expression to maintain cholesterol metabolism, we then sought to determine whether RBP4 influences SA receptor expression via CD36-mediated cholesterol uptake. We first investigated the impact of RBP4 expression on cholesterol uptake in HEK293T cells and murine bone marrow-derived macrophages (BMDMs) by using Dil-labeled OxLDL. This method enables tracking of cellular cholesterol uptake and localization through red fluorescence. Confocal microscopy analysis revealed that cholesterol uptake was significantly reduced in RBP4-deficient cells compared to control WT cells (Fig 5A and 5B), suggesting that RBP4 deficiency impairs cellular cholesterol uptake in different cell types. Moreover, flow cytometry analysis quantitatively confirmed that RBP4 contributes to cholesterol uptake (Fig 5C and 5D). Additionally, CD36 expression rescued the reduction of cholesterol uptake in RBP4-deficient cells (Fig 5E and 5F). These findings suggest that RBP4 is crucial for regulating cellular cholesterol uptake, likely through induction of the CD36 scavenger receptor.

Previous studies have demonstrated that an increase in cellular cholesterol content correlates with higher cholesterol levels in the cell membrane, leading to alterations in the quantity and distribution of SA receptors on the cell surface [37]. To assess whether CD36-mediated cholesterol changes affect SA receptor expression or distribution, we treated the CD36-overexpressing HEK293T cells and immortalized BMDMs with or without soluble cholesterol and examined SA receptor expression using by lectin-based flow cytometry. Cholesterol supplementation significantly elevated SA receptor expression, an effect potentiated by CD36 expression (Fig 5G and 5H). Confocal imaging confirmed that CD36-mediated cholesterol uptake increased SA receptor density at the plasma membrane without altering their spatial organization (Fig 5I). These results suggest that CD36-dependent cholesterol accumulation enhances SA expression on the cell surface. Collectively, these findings support the hypothesis that RBP4 promotes CD36 expression to regulate cellular cholesterol uptake, thereby modulating the expression of SA receptors that are critical for IAV attachment.

### Enforced expression of CD36 in RBP4-deficient cells enhances IAV infection

**Fig 6:**
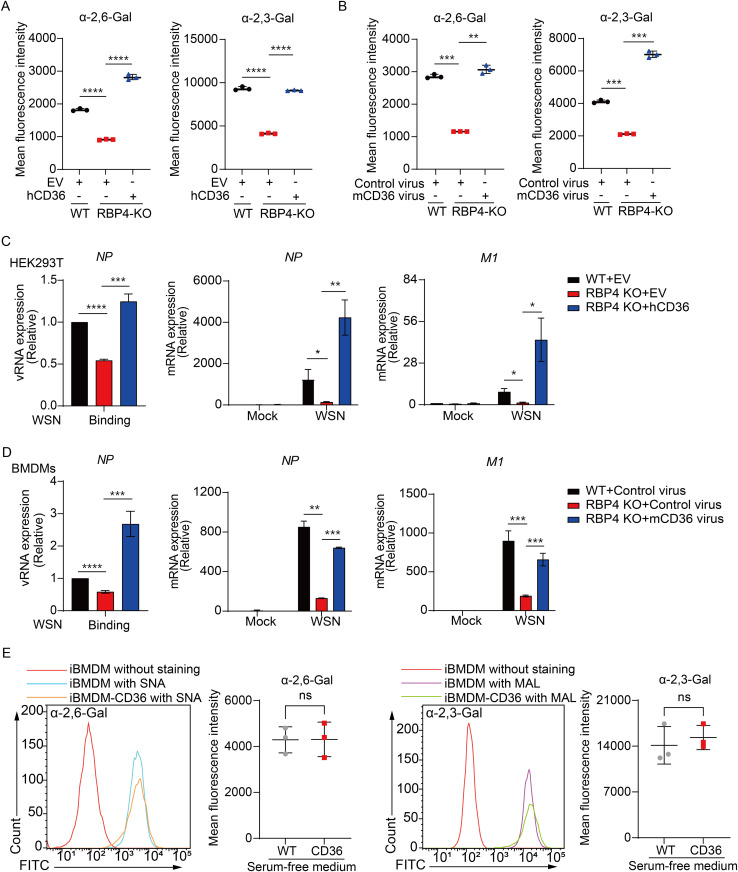
CD36 expression rescues IAV infection in RBP4-deficient cells. (A, B) Flow cytometry analysis of α-2,3-linked SA or α-2,6-linked SA in WT, RBP4-deficient and CD36 overexpressing RBP4-deficient HEK293T cells, as wells as murine BMDMs transduced with CD36 retrovirus. Mean fluorescence intensities were quantified with FlowJo software. (C, D) qPCR analysis of vRNA levels of the NP gene or NP and M1 mRNA expression in HEK293T cells (C) or BMDMs (D) infected with IAV WSN strain (MOI = 5) during the viral attachment stage. (E) Flow cytometry analysis of α-2,3-linked SA or α-2,6-linked SA in WT and stable CD36-overexpressing iBMDMs cultured in serum-free medium. Cells were cultured with serum-free medium for 6 hours and subsequently stained with Lectins. Mean fluorescence intensities were quantified with FlowJo software. Data are pooled from three independent experiments (A-E, mean ± SD). *p < 0.05, **p < 0.01, ***p < 0.001, ****p < 0.0001 (Student’s *t*-test).

To ascertain whether the attenuated virus infection in RBP4-deficient cells was due to lower levels of CD36, we investigated the impact of SA expression in these cells through CD36 overexpression. We found that the distribution of α-2,3-linked or α-2,6-linked SA in CD36-overexpressing RBP4-deficient HEK293T cells and RBP4-deficient BMDMs transduced with CD36 retroviruses was restored to levels comparable to those in WT cells (Fig 6A and 6B). These findings suggest that CD36-mediated cholesterol uptake is critical for modulating the expression or distribution of viral SA receptors on the cell surface. Consequently, CD36 expression promoted the binding of IAV in RBP4-deficient conditions, as evidenced by increased viral RNA expression (Fig 6C and 6D, left). Consistent with this, the mRNA levels of viral NP and M1 in RBP4-deficient cells were also restored to WT levels upon CD36 overexpression (Figs 6C and 6D, middle and right and S4D). To determine whether CD36 regulates sialic acid levels independently of cholesterol uptake, we cultured WT and CD36-overexpressing iBMDMs in serum-free medium, thereby depriving CD36 of its primary extracellular cholesterol source. Under these conditions, the α-2,6- and α-2,3-linked sialylation profiles were comparable in WT and CD36-overexpressing iBMDMs (Fig 6E), indicating that CD36 modulates sialic acid levels chiefly via its cholesterol-uptake function rather than through any cholesterol-independent mechanisms. Together, these data demonstrate that RBP4 facilitates IAV infection by orchestrating CD36 expression to mediate cholesterol uptake, thereby increasing the levels of SA receptor.

### Lentivirus-mediated overexpression of CD36 antagonized the diminished IAV replication and disease process in RBP4-deficient mice

**Fig 7:**
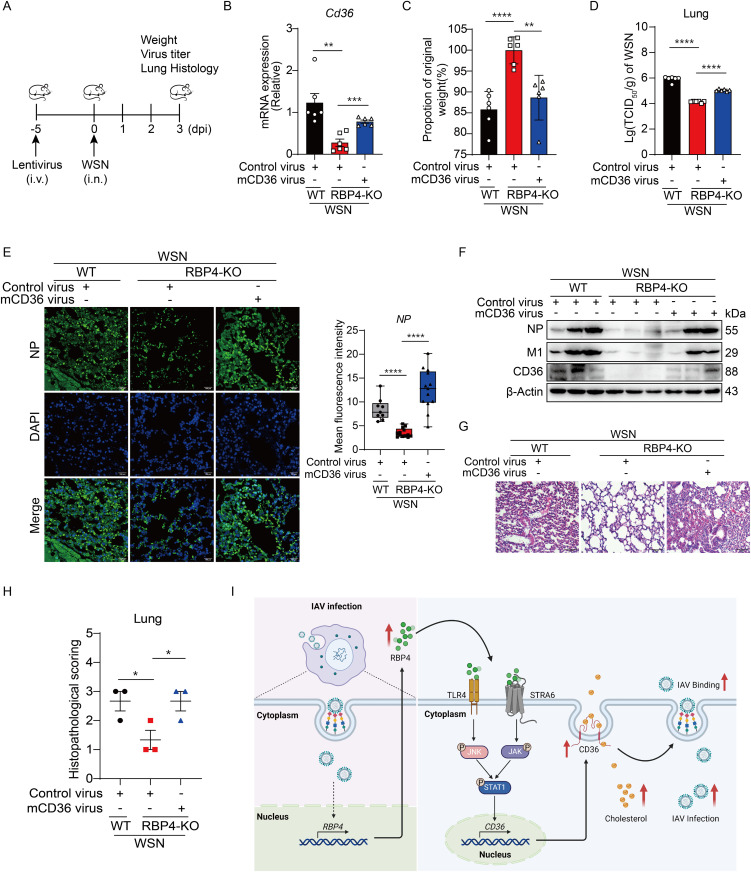
CD36 compromises host defense against IAV replication *in vivo.* (A) Experimental scheme for lentiviral transduction and influenza virus infection in mice. WT and RBP4-deficient mice were intravenously injected with lentivirus (10^9^ PFU in 200 μL per mouse) followed by infection with 10^4^ PFU of IAV (WSN strain) 5 days later. Lungs were collected 3 days post-IAV infection. (B) qPCR analysis of CD36 mRNA levels in lung tissues from WT, RBP4-deficient, and CD36-overexpressing RBP4-deficient mice as treated in (A). (C) Body weight changes of indicated mouse genotypes at day 3 post-IAV infection. (D) Plaque assay analysis of virus titer in lung tissues from mice described in (A). (E, F) Immunofluorescence images (E, left) and quantitative analysis of NP protein intensity (E, right) or immunoblotting analysis of M1, NP and CD36 protein expression (F) in lung tissues from the mice described in (A). Mean fluorescence intensity was determined by Image J software (E, right). Scale bars, 100 μm. (G, H) Representative H&E-stained lung sections (G) and corresponding histopathological scores (H) from indicated groups. Each dot in (B-D, H) represents an individual mouse. (I) Proposed model of RBP4-mediated promotion of influenza virus infection. Created in BioRender. Huang, L. (2025) https://BioRender.com/rll2ll1. Data are representative of two independent experiments (E-G). *p < 0.05, **p < 0.01, ***p < 0.001, ****p < 0.0001 (Student’s *t*-test).

To validate whether the aforementioned rescue phenotype of CD36 also occurs *in vivo*, we overexpressed murine CD36 via lentivirus infection in RBP4-deficient mice for five days and then challenged the mice with IAV infection for three days (Fig 7A). qPCR analysis revealed that the lentivirus-mediated CD36 was successfully overexpressed in the lungs of mice (Fig 7B), indicating that the experimental animal model was successful. Not surprisingly, CD36 overexpression exacerbated weight loss in RBP4-deficient mice (Fig 7C). Furthermore, plaque assay analysis demonstrated that RBP4 deficiency attenuated influenza virus titers in the lungs, while exogenous expression of CD36 rescued this attenuation in RBP4-deficient mice infected with IAV (Fig 7D). These data suggest that CD36 expression can rescue the attenuated influenza virus replication or infection in RBP4-deficient conditions *in vivo*. Consequently, the expression of NP and M1 protein significantly rebounded after CD36 overexpression in the lungs of RBP4-deficient mice (Fig 7E and 7F). Meanwhile, HE staining showed that RBP4 deficiency mitigated lung injury in mice compared to WT mice, while CD36 overexpression intensified lung injury and histological scores (Fig 7G and 7H). Taken together, these results demonstrate that RBP4 promotes influenza virus replication and accelerates disease processes *in vivo* by regulating CD36 expression (Fig 7I).

## Discussion

Influenza A virus (IAV) poses a significant public health threat. While numerous factors have been identified as contributing to influenza virus infection, the key host-pathogen interactions regulating IAV replication are not fully understood. In this study, we identified RBP4, a member of the lipocalin family responsible for retinol transport, as a crucial host factor that facilitates influenza virus infection during the early stage of the viral life cycle. IAV infection induces the expression of RBP4 in both cells and patients. Overexpression of RBP4 promotes IAV infection while its deficiency suppresses infection by the human influenza virus H1N1, swine influenza virus H3N2, and avian influenza virus H9N2, both *in vitro* and *in vivo*. Specifically, secreted RBP4 activates STAT1 signaling through binding to its receptors TLR4 or STRA6, leading to increased expression of CD36, a scavenger receptor involved in lipid metabolism and cholesterol uptake. Consequently, the distribution of IAV membrane sialic acid receptors on the cell surface is increased, enhancing IAV attachment [38]. Therefore, our findings, combined with the previous studies [10], established that cholesterol homeostasis plays a critical role in regulating IAV infection.

RBP4 plays a decisive role in various physiological processes, including insulin sensitivity, lipid metabolism, and inflammatory responses [39]. Its function can be dependent on its role in retinoid homeostasis or independent of retinol transport. Notably, recent studies have shown strong correlations between RBP4 levels and infection by multiple viruses, yet the exact roles of RBP4 in virus infection remain unclear [22,40]. Previous studies have shown that RBP4 levels increase during HCV infection, and knockdown of RBP4 increases HCV replication [41], indicating that RBP4 expression inhibits HCV replication. Our recent study also suggests that RBP4 limits PCV2 infection through activation of the selective autophagy [23]. Moreover, RBP4 expression is induced *in vitro* or *in vivo* in response to infection by several RNA viruses, including human immunodeficiency virus and IAV [24,42]. Here, we found that RBP4 facilitates IAV infection by modulating cellular cholesterol homeostasis, adding a mechanism by which RBP4 promotes virus infection. It is noteworthy that RBP4 levels are significantly elevated in the serum of patients infected with IAV [24]. Hence, RBP4 levels appear to modulate IAV-driven pathogenesis, and suppression of RBP4 could attenuate IAV replication or infection. Given that IAV infection activates several signaling pathways-most notably NF-κB and MAPKs, the virus may control RBP4 transcription through these pathways. Additional studies are required to elucidate the precise mechanisms underlying IAV-mediated induction of RBP4.

Influenza virus infects cells by binding to sialylated glycans on the cell surface. The virus attaches to SA receptors located in regions of cell membrane, enriched in cholesterol and sphingolipids [43]. Because RBP4 does not affect IFN production and expression of downstream ISGs [23], we then focused on whether RBP4 regulates critical stages in the IAV life cycle. We found that RBP4 expression facilitates the binding of IAV to multiple types of host cells. In fact, deficiency of RBP4 suppresses the expression of SA receptors on the cell surface. Interestingly, cholesterol accumulation was also reduced in RBP4-deficient cells. This observation is supported by the positive correlation of RBP4 levels and total cholesterol and triglycerides in the plasma of clinical patients [44], suggesting that RBP4 may be involved in cholesterol homeostasis. Notably, the local cholesterol concentration in the plasma membrane of cells may enhance influenza binding avidity by controlling receptor clustering [37]. Therefore, our findings indicate that RBP4 may modulate SA distribution on the cell surface through regulation of cholesterol homeostasis. It is well known that the cell membrane contains 60–80% of the total cellular cholesterol [45], which is vital for proper cellular and system functions [29]. The cellular cholesterol level reflects the dynamic balance between biosynthesis, uptake and other regulatory processes [46]. In our study, the cholesterol uptake receptor CD36 is significantly reduced upon RBP4 deficiency, both *in vitro* and *in vivo*, indicating that RBP4 expression elevates the expression of CD36 to mediate cholesterol uptake. This notion is further supported by the finding that overexpression of CD36 enhances the uptake of soluble cholesterol and leads to an increase of cell surface SA. Hence, our data provide further mechanistic insights into the role of RPB4 in cholesterol homeostasis and how this affects IAV binding on host cells. Further studies are required to elucidate how RBP4 modulates SA surface expression through cholesterol alterations [37,47], and to determine whether RBP4 influences the budding process of IAV [48]. However, it is noteworthy that RBP4 deficiency enhanced SeV replication while suppressing that of VSV. Given that SeV strictly depends α-2,3-linked sialic acids for entry whereas VSV uses non-sialic acid receptors [26,27], these opposing phenotypes indicate that RBP4 may modulate post-entry steps of viral infection through sialic-acid-independent mechanisms, highlighting its multifaceted role in virus–host interactions.

CD36 is a type 2 cell surface scavenger widely expressed in many immune and non-immune cells. It functions as both free acid (FA) transporter and signaling receptor responding to extracellular signals such as danger- or pathogen- associated molecular patterns (DAMPs and PAMPs) [49]. Notably, CD36 localizes preferentially in cholesterol-rich membrane lipid rafts. Thus, as a fatty acid transporter, CD36 binds to long-chain fatty acids and modulates oxLDL uptake and cellular cholesterol levels. The expression of CD36 is regulated at both transcriptional and posttranslational levels, but regulation differs among different cell types. Interestingly, it has been shown that RBP4 activates of the c-Jun N-terminal kinase (JNK) and signal transducer and activator of transcription (STAT) 1 pathway in a TLR4-dependent manner to mediate CD36 transcription [32], as macrophages do not express STRA6 receptor. Consistently, we also found that CD36 transcription was induced by RBP4 expression through TLR4 activation in porcine alveolar macrophages, highlighting the importance of RBP4 in regulation of CD36 expression, as well as lipid metabolism in immune cells. In fact, induction of CD36 expression by various transcription factors is generally accompanied by an increase in lipid or lipoprotein uptake [50]. In non-immune cells, however, whether RBP4 regulates CD36 transcription through activation of JNK-STAT1 signaling remains unclear. Interestingly, we found that deficiency of RBP4 still reduced CD36 expression in HEK293T cells, which are commonly used as a cellular model for studying influenza virus without expressing TLRs. These results suggest that RBP4 may also regulate the transcription of CD36 in a TLR-independent way. Indeed, knockdown of STRA6 impaired STAT1 activation induced by RBP4 in HEK293T cells, leading to suppression of CD36 expression. This data is further strengthened by the finding that JAK inhibition eliminated STAT1 activation as well as CD36 expression in HEK293T cells. Our data thus support the previous study showing that RBP4 induces the phosphorylation of JAK2, increases the expression of STAT target genes in a STRA6-depenent manner. Although IAV infection itself may also activate JNK and STAT1, it is important to highlight that RBP4 deficiency impairs IAV attachment with the first 2 hours, underscoring the critical role of RBP4-mediated cholesterol modulation in enhancing IAV binding avidity. Therefore, our findings provide evidence that RBP4 regulate CD36 transcription via STAT1 activation in both TLR- or STRA6-dependent manners and support the notion that the regulation of CD36 expression differs among distinct cell types.

In clinical, obese patients are at a higher risk of requiring intensive care and experiencing longer durations of mechanical ventilation due to influenza compared to non-obese patients [51,52]. Additionally, RBP4 levels are elevated in the serum and adipose tissue of individuals with obesity-induced insulin resistance and are associated with other metabolic syndromes [16]. This aligns with our findings that increased cholesterol accumulation enhances IAV infection. It is plausible that elevated RBP4 levels in obesity may promote cholesterol uptake, disrupt lipid homeostasis [53], and thereby facilitate influenza virus infection, potentially leading to disease progression. Thus, the role of RBP4 in modulating host cholesterol metabolism to enhance influenza virus infection may have significant clinical implications.

In summary, we have identified RBP4 as a pivotal host factor that facilitates influenza virus infection and established that RBP4 acts as a catalyst for IAV infection through modulating cellular cholesterol homeostasis.

## Materials and methods

### Ethics statement

All animal experimental protocols were reviewed and approved by the Shandong Agricultural University Animal Care and Use Committee (Approval Number: # SDAUA-2018–057) and were performed in strict compliance with the Animal Ethics Procedures and Guidelines of the People’s Republic of China.

### Data acquisition and processing

The datasets (GSE155635, GSE158592, GSE196350 and GSE213168) related to influenza virus infection was retrieved from the GEO database (http://www.ncbi.nim.nih.gov/geo/) and screened for normal samples and disease samples separately. Subsequently, the unpaired *t*-test differential analysis of RBP4 expression in the blood of healthy people and patients from the screened datasets were performed by GraphPad Prism (v8.0).

### RNA sequencing (RNA-Seq) analysis

For RNA-seq analysis, BMDMs from WT and RBP4-KO mice were then harvested for total RNA extraction with a total RNA purification kit (Promega). RNA quality was analyzed by Agilent 2100 Bioanalyzer (Agilent Technologies, CA, USA). 1 mg of total RNA with RNA integrity number (RIN) value above 9 in this study was converted into RNA-seq quantification libraries. 1 μg total RNA was used for following library preparation. Next generation sequencing library preparations were constructed according to the manufacturer’s protocol. Pair end RNA-seq reads were aligned to mouse genome mm10 via software Hisat2 (v2.0.1) and only uniquely mapped reads were preserved. For coverage of mapped RNA-seq reads in transcripts, the expression level of each genes transcripts was calculated as the normalized fragment count to fragments (FPKM). After adding an offset of 1, FPKM data were log-transformed for the analyses in this study [54]. Genes with adjust Log_2_FC≥0.2 between two conditions were regarded as significantly up-regulated genes, and significantly down-regulated genes were identified with Log_2_FC ≤ -0.2 between two conditions. Heatmap was generated with TBtools software (2.119). The RNA-seq data reported in this study have been deposited in the Gene Expression Omnibus with the accession number GSE290075.

### Cells and viruses

Human lung epithelial cells (A549), human embryonic kidney 293T cells (HEK293T) cells, immortalized BMDMs (iBMDMs), PK-15 cells, 3D4/21 cells were cultured in Dulbecco’s modified Eagle’s medium (DMEM) supplemented with 10% (v/v) fetal bovine serum (FBS, Sigma, USA) and 1% penicillin-streptomycin (Gibco, USA). RBP4-deficient HEK293T and PK-15 cells were generated by CRISPR-Cas9 gene editing as previously described [23], and the target genomic loci were sequenced by Sangon Biotech (Shanghai, China) for knockout verification. Murine bone marrow-derived macrophages (BMDMs) from WT or RBP4-deficient mice were obtained and maintained as previously described in DMEM supplemented with 10% fetal bovine serum (FBS, Gibco, USA) and 10% L929 cell supernatant as conditioned medium providing macrophage colony stimulating factor. All cells were incubated at 37 °C in a humidified atmosphere containing 5% CO_2_.

The IAV strains used were WSN (A/WSN/1933, H1N1), PR8 (A/Puerto Rico/8/1934, H1N1), swine-origin H3N2 (A/swine/Shandong/ZYZ/2021), and avian-origin H9N2 (LPAI, A/chicken/Henan/F1112/2020) in this study. The viruses were amplified using 10-day-old embryonic chicken eggs with specific-pathogen-free (SPF) and then titrated by using plague assays on MDCK cells. All experiments with IAV infection were performed in a biosafety level 2 laboratory according to the protocol.

### Mice

RBP4-deficient mice were obtained from the Kumamoto University (Honjo, Kumamoto, Japan) on a C57BL/6J genetic background. Mice were maintained in SPF barrier facilities and were bred to produce both WT and RBP4-deficient mice. Mice genotypes were identified by PCR assays with specific primers (GSP-F: 5’- CTCGGCTCCGTCGCTCCACG-3’, GSP-R: 5’-CCAGAGCCCAGA GAACTGAG-3’, and mPGK-R: 5’- TACCCGCTTCCATTGCTCAG-3’). Six-week-old mice were used for all experiments.

### Antibodies and reagents

The primary antibodies used in this study were sourced from commercial suppliers as follows: Rabbit polyclonal antibodies against RBP4 (1 : 2,000, 11774–1-AP), CD36 (1 : 2,000, 18836–1-AP), STRA6 (1 : 3,000, 22001–1-AP), p65 (1 : 1,000, 10745–1-AP) and β-actin (1 : 20,000, 66009–1-lg) were purchased from Proteintech Group Inc. Rabbit polyclonal antibodies against CD36 (1 : 1,000, CY5796) and FLAG (1 : 10,000, AB0030) were purchased from Abways. Antibody against p38α/β (1 : 1,000, sc-7972) was purchased from Santa Cruz Biotechnology. Antibodies against SPAK/JNK (1 : 1,000, 9252), phospho-SPAK/JNK (Thr183/Tyr185) (1 : 2,000, 9255), STAT1 (1 : 1,000, 14994), phospho-STAT1 (Tyr701) (1 : 1,000, 9167), JAK2 (D2E12) (1 : 1,000, 3230), phosphor-JAK2 (Tyr1007/1008) (C80C3) (1 : 1,000, 3776), mTOR (1 : 1,000, 2983), phosphor-mTOR (1 : 1,000, 5536), phosphor-p65 (1 : 1,000, 3033), phosphor-p38 (1 : 1,000, 9215), ERK1/2 (1 : 1,000, 9102), phosphor-ERK1/2 (1 : 1,000, 9101) were purchased from Cell Signaling Technology. Mouse anti-M1 monoclonal antibody (1 : 5,000) and rabbit anti-NP polyclonal antibody (1 : 5,000) were generated previously [55]. Porcine RBP4 recombinant protein was yielded as previously described [56].

### Inhibition of signaling pathways

For chemical inhibitor assays, cells were pretreated with following chemical inhibitors for 2 hours before the addition of recombinant RBP4 protein (80 μg/mL) for 24 hours before analysis. The following chemical inhibitors including JNK inhibitor SP600125 (10 μM, HY-12041), JAK inhibitor Ruxolitinib (5 μM, HY-50856), TLR4 inhibitor Resatorvid (1 μM, HY-11109), p38 inhibitor SB203580 (10 μM, HY-10256), NF-κB inhibitor BAY11–7082 (10 μM, HY-13453), MEK inhibitor U0126 (10 μM, HY-12031A) and mTOR inhibitor Rapamycin (10 μM, HY-10219) were used in the study. All chemical inhibitors were purchased from Med Chem Express (MCE, New Jersey, USA).

### Plasmids and transfection

Porcine RBP4 was previously constructed in the laboratory [23]. Human RBP4 was amplified by PrimeSTAR HS DNA Polymerase (Takara Bio, Beijing, China) with cDNA of A549 cells as a template, followed by *Hind* Ⅰ and *Xho* Ⅰ digestion, and was ligated into the pcDNA3.0-Flag vector (Addgene). Human CD36 was amplified by PrimeSTAR HS DNA Polymerase with complementary DNA (cDNA) of HEK293T cells as a template, followed by *EcoR* Ⅰ and *Xho* Ⅰ digestion, and was ligated into the pCMV-Myc vector (Addgene). Primers used to construct these expression vectors are listed in S1 Table. All constructs followed standard molecular cloning protocols and were then sequenced. Protein expression of the constructs was further confirmed by immunoblotting. Plasmids were transfected into HEK293T, PK-15, 3D4/21 or A549 cells using Lipofectamine 2000 reagent (Invitrogen, USA) according to the manufacturer’s instructions at a final concentration of 1 μg.

### RNA interference

Small interfering RNAs (siRNA) specifically targeting human STRA6, human RBP4 and a non-targeting control siRNA were previously reported [57] or were designed by Shanghai GenePharma Co., Ltd., China. All siRNA oligos were synthesized by Shanghai GenePharma Co., Ltd., China, and were transfected into HEK293T or A549 cells using Lipofectamine RNAiMAX reagent (Thermo Fisher Scientific, USA) according to the manufacturer’s instructions. The human STRA6 and RBP4 siRNA target sequences are listed as follows: siSTRA6-sense (human): 5′-GUCUACAUCCUCCCUCUCA-3′; siSTRA6-antisense (human):5′-UGAGAGGGAGGAUGUAGAC-3′; siRBP4-sense (human): 5′- CGCAGAAGAUUGUAAGGCATT-3′; siRBP4-antisense (human):5′- UGCCUUACAAUCUUCUGCGTT-3′.

### Virus infection

For IAV infection *in vitro*, cells were infected with different strains of IAV, including H1N1 virus (WSN and PR8 strains), swine H3N2 virus or avian H9N2 virus, at various multiplicity of infection (MOI) as indicated. After 1-hour incubation period at 37°C and 5% CO_2_, cells were washed twice with PBS to remove unbound virus. Subsequently, the wells were refilled with complete medium containing 0.2% bovine serum albumin (BSA) (Sigma-Aldrich, USA) and 0.1 μg/mL tosyl-phenylalanine chloromethyl-ketone (TPCK)-treated trypsin (Sigma-Aldrich, USA) to facilitate virus entry. For IAV infection *in vivo*, mice were anesthetized by intraperitoneal injection of tribromoethanol at a dose of 400 mg/kg. Following anesthesia, mice were inoculated intranasally with 10^4^ plaque-forming units (PFU) of the WSN strain in a volume of 50 μL. Mice were then monitored daily for weight loss and general health over a period of 6 days. At 3 days post-infection, mice were euthanized, and tissues were harvested for further analysis. SeV and VSV infections were performed as previously described [58].

### Plaque assay

Plaque assay was generally performed as previously described [59]. In brief, MDCK cells were seeded in 12-well plates and incubated at 37 °C with 5% CO_2_ until they reached approximately 80–90% confluence. The cells were then washed twice with PBS to remove any residual medium. Following the wash, cells were incubated with serial 10-fold dilutions of IAV for 1 hour with swirling every 15 minutes. The culture supernatant was then aspirated, and the cells were overlaid with minimum essential medium (Invitrogen, USA) containing 1% low-melting agarose, 0.4% BSA, 1% penicillin/streptomycin, and TPCK-trypsin at a final concentration of 1.25 μg/mL. At 72 hours post infection, the agarose layer was carefully removed and the cells were fixed with 10% formalin for 1 hour at room temperature. The plaques were eventually visualized with 0.1% crystal violet solution.

### Histopathology

Histopathological analysis was performed as previously described [60]. In brief, lung tissues from mice were fixed in 10% neutral buffered formalin overnight, trimmed, dehydrated, and embedded in paraffin. Sections were cut into 5 μm and stained with hematoxylin-eosin (H&E). Images were acquired on an Olympus microscope (CX41RF) using imaging software (MiE V3.1). Histological score was adapted from previously described [61].

### Immunoblotting analysis

Whole-cells lysates were prepared as described previously [62]. Protein samples were separated by 10% sodium dodecyl sulfate-polyacrylamide gel electrophoresis (SDS-PAGE), and transferred to polyvinylidene fluorid membranes (Millipore, USA) by electroblotting. The membranes were blocked with 5% (w/v) skim milk in PBS containing 0.1% Tween 20 for 2 hours at room temperature and then were incubated with the appropriately diluted primary antibodies in PBS containing 2.5% BSA overnight at 4 °C. Subsequently, the membrane was incubated with secondary antibodies and the blots visualized by using an auto chemiluminescent imaging system (Tanon, China) according to the manufacturer’s instructions.

### Reverse transcription and quantitative real-time PCR (qPCR)

Total RNA was extracted from cells using the SV Total RNA Isolation System (Promega, USA) and was reversely transcribed to cDNA using M-MLV reverse transcriptase with RNase inhibitor (Takara Bio, China). An oligo(dT)_20_ primer was used for RT of cellular mRNA. The primer for reverse transcription (RT) of influenza virus RNA was previously reported [63]. qPCR was performed in triplicated determinants with RealStar Green Fast Mixture (GenStar, China) on a StepOne plus thermal cycler (ABI, USA). Threshold cycle values were normalized to triplicated samples amplified with primers specific for glyceraldehyde-3-phosphate dehydrogenase (GAPDH). The primer sequences used for qPCR are listed in S1 Table.

### Virus attachment and internalization assay

Cells were seeded in 12-well plate and cultured for 16–20 hours, then were incubated with IAV, SeV or VSV at an MOI = 5 for 1 hour at 4 °C. For the virus attachment assay, cells were washed three times with chilled PBS to remove unbound virus and harvested to determine the amount of viral RNA by qPCR. For the internalization assay, after the initial viral binding, cells were washed three times with ice-cold PBS to remove the unbounded viruses and filled with fresh culture medium. The culture temperature of cells was then shifted to 37 °C for 30 min to allow virus internalization. Following internalization period, the cells were then washed three times with ice-cold PBS-HCl (pH = 1.3) to remove the virus particles that were attached but not internalized. Subsequently, total cellular RNA was extracted to analyze viral RNA by using qPCR.

### Confocal microscopy

For indirect immunofluorescence, cells were fixed with 4% paraformaldehyde (PFA) for 20 min at room temperature (RT), permeabilized with 0.1% TrixtonX-100 for 10 min, and blocked with 5% BSA (Sigma-Aldrich, USA) for 30 min. Cells were then incubated with the appropriate primary antibodies diluted in PBS 2.5% BSA overnight at 4°C in a humidified chamber. After incubation with the primary antibodies, cells were stained with Alexa Fluor 488- or 594- conjugated secondary antibodies for 60 min. For the lectin staining, cells were fixed with 4% PFA for 20 min at RT after removal of different lectins (MAL, SNA, Dil-OxLDL) and were stained with 4’,6-diamidino-2-phenylindole (DAPI, C1005, Beyotime, China) or 1,1’-dioctadecyl-3,3,3’,3’-tetramethylindocarbocyanine perchlorate (Dil, C1036, Beyotime, China) to label cell nucleus or membrane, respectively. For immunofluorescence with tissues, sections were subjected to antigen retrieval before incubation with primary antibody. Images were captured with a laser scanning confocal microscope (Andorra, UK) with Fusion software. The fluorescence intensity was analyzed by ImageJ software (NIH, 1.8.0, USA).

### Flow cytometry analysis

Sialic acid (SA) on the cell surface detection was performed using flow cytometry as previously described [64]. Briefly, cells were labeled with biotinylated Maackia amurensis lectin (MAL, 10 μg/mL) for α-2,3-SA or Sambucus nigra lectin (SNA, 5 μg/mL) for α-2,6-SA (Vector Laboratories, USA). After staining for 40 min at 4°C, cells were washed three times to remove any unbound lectin, and were resuspended with chilled PBS for measurement of fluorescence intensity using a FACSAria flow cytometer (BD Biosciences, USA). Untreated cells served as negative controls. Data were analyzed using FlowJo software (Flow Jo, 10.8.1, USA).

### Total cholesterol measurement and cholesterol uptake assay

Total cellular cholesterol level was quantified using a colorimetric cholesterol assay kit obtained from Nanjing Jiancheng Bioengineering Institute (A111-1–1, Nanjing, China) according to the manufacture’s protocol. Briefly, cells were harvested and lysed using 1% Triton X-100, and an aliquot of 3 μL of the lysate was used for each assay reaction. Samples were pipetted into separate wells of a microplate followed by adding 300 μL of the working solution. After 10 min of incubation at 37°C in dark, the fluorescence intensity was measured in a fluorescence microplate reader with a filter set for excitation at 500 nm (BioTek, USA). The fluorescence readings were then correlated to a standard curve to calculate the total cholesterol content within the cells. Cholesterol uptake assays were performed as previously described [32]. HEK293T cells or BMDMs in 12-well plates were first incubated with 10 μg/mL Dil-labeled oxidized low-density lipoprotein (Dil-OxLDL) for 4 hours at 37 °C. Cells were then washed three times with PBS and fluorescence intensity was analyzed using confocal microscope or flow cytometry.

### Dual-luciferase reporter assay

To assess the activity of the human CD36 (hCD36) promoter, a reporter plasmid was generated by inserting CD36 promoter sequence containing the STAT1 binding site, ranging from positions -724 to +83 nucleotides relative to the transcription start site, into pGL3-basic vector [65]. HEK293T were co-transfected in duplicates with the pGL3-hCD36 plasmid along with the human RBP4-expressing plasmid or a control vector using Lipofectamine 2000 reagent (Invitrogen, USA) for 24 hours. A renilla luciferase reporter gene (pRL-TK, Promega) was co-transfected to serve as an internal control. Following the transfection period, cells were then harvested and cell lysates were prepared for analysis using a Dual-Luciferase Report Assay System (Promega, USA) according to the manufacture’s protocol.

### Virus packaging and transduction

Mouse CD36 ORF were cloned into the pMX-puro vector for retrovirus transduction or into the pCDH-CMV-EF1a-Puro vector for lentiviral packaging. Retroviral transduction was performed as previously described [66]. Briefly, Plat-E cells (4 × 10^6^) were plated into 10 cm dishes and cultured for 24 hours. Cells were then transfected with 15 µg of pMX-Puro-CD36 or control retroviral vectors using Lipofectamine 2000 (Invitrogen, USA). After 48 hours, the viral supernatants were collected, filtered, and 5 mL of viral supernatant was used to transduce 5 × 10^6^ BMDMs or iBMDMs in the presence of 6 µg/mL of polybrene (Solarbio, China) for 24 hours. Overexpression of CD36 in BMDMs or iBMDMs were achieved by selection with 2 µg/mL puromycin (Solarbio, China) for 3 days. For lentiviral packing, Lenti-X 293T cells were seeded in 10 cm dishes and co-transfected with pVSVG (3 μg), psPAX.2 (5 μg), and pCDH-mCD36-CMV-EF1a-Puro (7 μg), or empty vector using Lipofectamine 2000. The medium was replaced with fresh medium 6 hours post-transfection. Viral supernatants were collected at 48 and 72 hours, and then concentrated using a 5 × lentivirus concentration solution (Applygen, Beijing, China, E2055) for subsequent titration and establishment of the overexpression mouse system.

### Statistics

Statistical analysis was performed using GraphPad Prism (v8.0) (GraphPad, USA). Significance was determined using two-tailed Student’s *t*-test or One-way ANOVA. *p* value ≤ 0.05 were considered statistical significance.

## Supporting information

S1 Fig IAV infection actives multiple signaling cascades to regulate RBP4 transcription. (A) qPCR analysis of RBP4 mRNA levels in HEK293T cells upon WSN (MOI = 5) infection at indicated periods. (B) Schematic diagram of putative transcription factor (TF) binding sites on *RBP4* promoter. (C) Immunoblotting analysis of RBP4, phosphorylated (p-) and total p65, p-p38 and p38, p-ERK1/2 and ERK1/2, and p-mTOR and mTOR protein levels in HEK293T cells upon WSN (MOI = 5) infection at indicated periods. (D) Immunoblotting analysis of RBP4, p-p65 and p65, p-p38 and p38, p-ERK1/2 and ERK1/2, and p-mTOR and mTOR protein levels in whole lysates of A549 cells pretreated with DMSO or indicated chemical inhibitors for 3 hours, followed by WSN (MOI = 5) infection for 6 hours.
(TIF)

S2 Fig RBP4 deficiency impairs early-stage IAV replication cycle. (A) Immunoblotting analysis of M1 protein levels in A549 cells transfected with RBP4 or control plasmids for 12 hours, following infection with WSN (MOI = 0.1) for indicated periods. (B, C) Sequencing validation (B) and immunoblotting analysis (C) confirming RBP4 knockout in clonal HEK293T and PK-15 cell lines. (D) qPCR or immunoblotting analysis of RBP4 mRNA or protein expression in A549 cells transfected with siRNA for RBP4. (E) qPCR analysis of mRNA levels of M1 and NP (left and middle) in A549 cells transfected as in (D) and then infected with WSN (MOI = 0.1) for the indicated periods. Viral titers (right) were determined by plaque assay at 24 hours post infection. (F) Immunoblotting analysis of RBP4 protein expression in WT and RBP4-deficient BMDMs. (G) Immunoblotting analysis of M1 protein levels in the lungs from WT and RBP4-deficient mice 3 days post-H9N2 IAV infection. Numbers indicated individual mice. (H, I) qPCR (H) or immunoblotting (I) analysis of NP and M1 mRNA and protein levels in WT and RBP4-defecient HEK293T cells infected with WSN strain (MOI = 5) for 3 hours. (J) Immunofluorescence staining of NP proteins (green) in WSN-infected (MOI = 5, 3 hours) WT and RBP4-deficient PK-15 cells. Nuclei were counterstained with DAPI (blue). Right panel shows mean fluorescence intensity from three independent experiments. Scale bars, 10 μm. (K) qPCR analysis of NP vRNA levels in RBP4-deficient PK-15 cells infected with WSN strain (MOI = 5) at the attachment stage, following treatment with recombinant porcine RBP4 (rpRBP4). (L) qPCR analysis of vRNA levels of SeV (left) or VSV (right) in WT and RBP4-deficient HEK293T cells infected with SeV (MOI = 5) or VSV (MOI = 5) for attachment assay. (M) qPCR analysis of L mRNA expression (left) in WT and RBP4-deficient HEK293T infected with SeV (MOI = 1), or N mRNA expression (right) in WT and RBP4-deficient PK-15 cells infected with VSV (MOI = 0.1) for indicated periods. Data are pooled from three independent experiments (D, E, K-M, mean ± SD). *p* values were calculated using unpaired student’s *t*-test. *p < 0.05, **p < 0.01, ***p < 0.001, ****p < 0.0001.
(TIF)

S3 Fig RBP4 upregulates CD36 expression through STAT1 activation. (A) qPCR analysis of selected differential genes (from the heatmap in Fig 4C) in BMDMs from WT and RBP4-deficient mice. (B) qPCR analysis of HMGCR, DHCR24 and SR-A mRNA levels in WT and RBP4-KO HEK293T cells. (C, D) Quantification of p-JNK, p-STAT1 and CD36 protein levels (normalized to total JNK, STAT1 or β-Actin, respectively) as in Fig 4H and 4I. Data were pooled from three independent experiments (A and B). *p < 0.05, **p < 0.01, ***p < 0.001, ****p < 0.0001, ns, not significant (Student’s *t*-test).
(TIF)

S4 Fig RBP4 regulates CD36 expression via STAT1 activation. (A) qPCR analysis of STRA6 and TLR4 mRNA levels in PK-15, HEK293T, BMDMs, and 3D4/21 cells. (B) Immunoblotting analysis of p-STAT1 protein levels in WT and RBP4-deficient HEK293T cells infected with WSN (MOI = 0.1) at indicated periods. (C) Luciferase reporter assay in HEK293T cells co-transfected with hCD36 promoter-driven reporter plasmid and either an RBP4 expression plasmid or empty vector. Luciferase activity was measured 24 hours post-transfection. (D) Immunoblotting analysis of CD36 protein levels in WT, RBP4-deficient and CD36 overexpressing RBP4-deficient HEK293T cells, as well as murine CD36 retrovirus-transduced BMDMs. Data are pooled from three independent experiments (C, mean ± SD). *p < 0.05 (Student’s *t*-test).
(TIF)

S1 Table Primers sequences used in this study. (DOCX)
