# Supplementary figures and images for: Retinol binding protein 4 enhances cellular cholesterol uptake to facilitate influenza A virus infection
Source: PLoS Pathog. 2025 Oct 27;21(10):e1013623. doi: 10.1371/journal.ppat.1013623 (PMC12558542; doi:10.1371/journal.ppat.1013623)

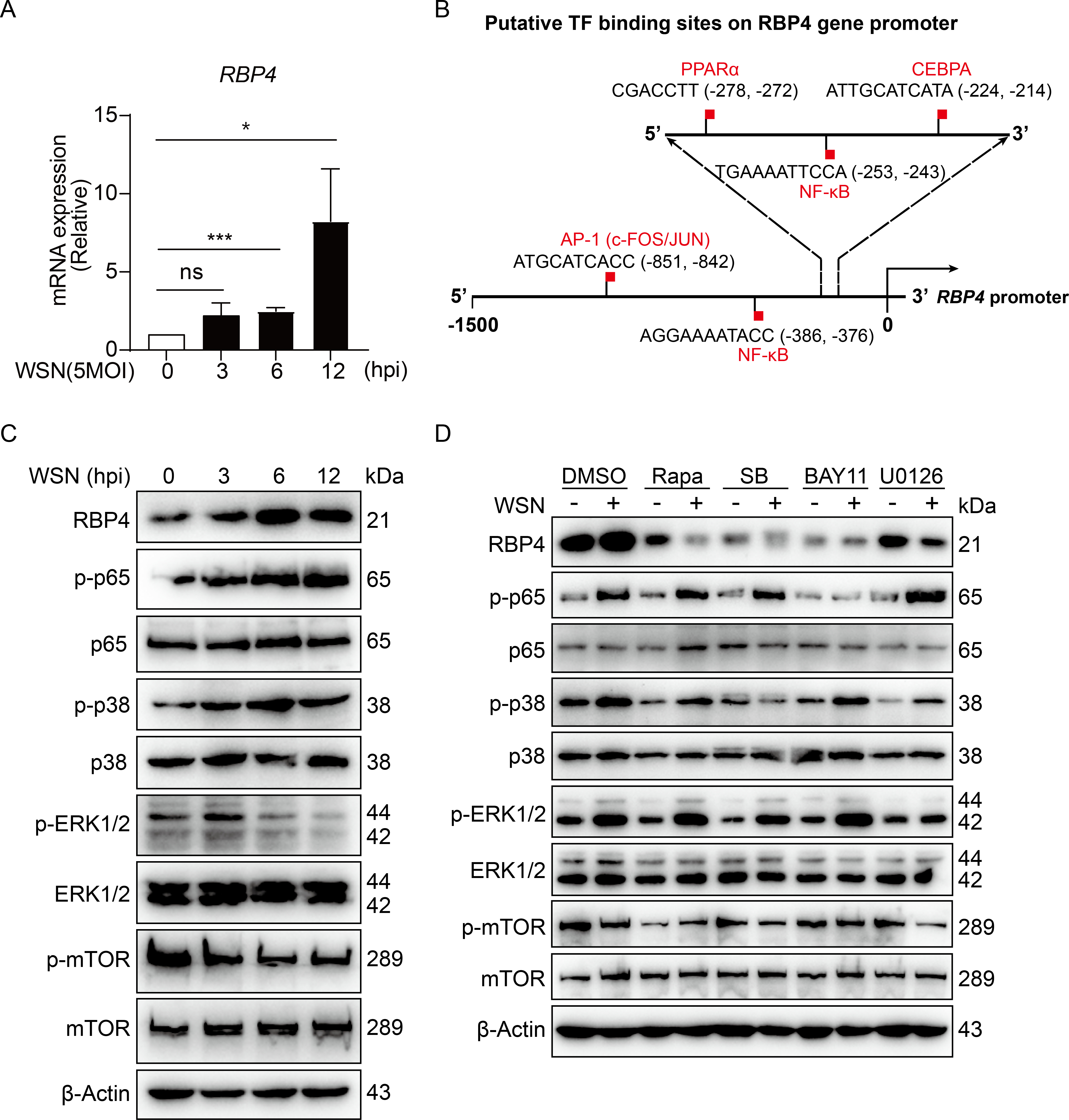

Supplement: S1 Fig — (A) qPCR analysis of RBP4 mRNA levels in HEK293T cells upon WSN (MOI = 5) infection at indicated periods. (B) Schematic diagram of putative transcription factor (TF) binding sites on RBP4 promoter. (C) Immunoblotting analysis of RBP4, phosphorylated (p-) and total p65, p-p38 and p38, p-ERK1/2 and ERK1/2, and p-mTOR and mTOR protein levels in HEK293T cells upon WSN (MOI = 5) infection at indicated periods. (D) Immunoblotting analysis of RBP4, p-p65 and p65, p-p38 and p38, p-ERK1/2 and ERK1/2, and p-mTOR and mTOR protein levels in whole lysates of A549 cells pretreated with DMSO or indicated chemical inhibitors for 3 hours, followed by WSN (MOI = 5) infection for 6 hours. (TIF) [file ppat.1013623.s001.tif]

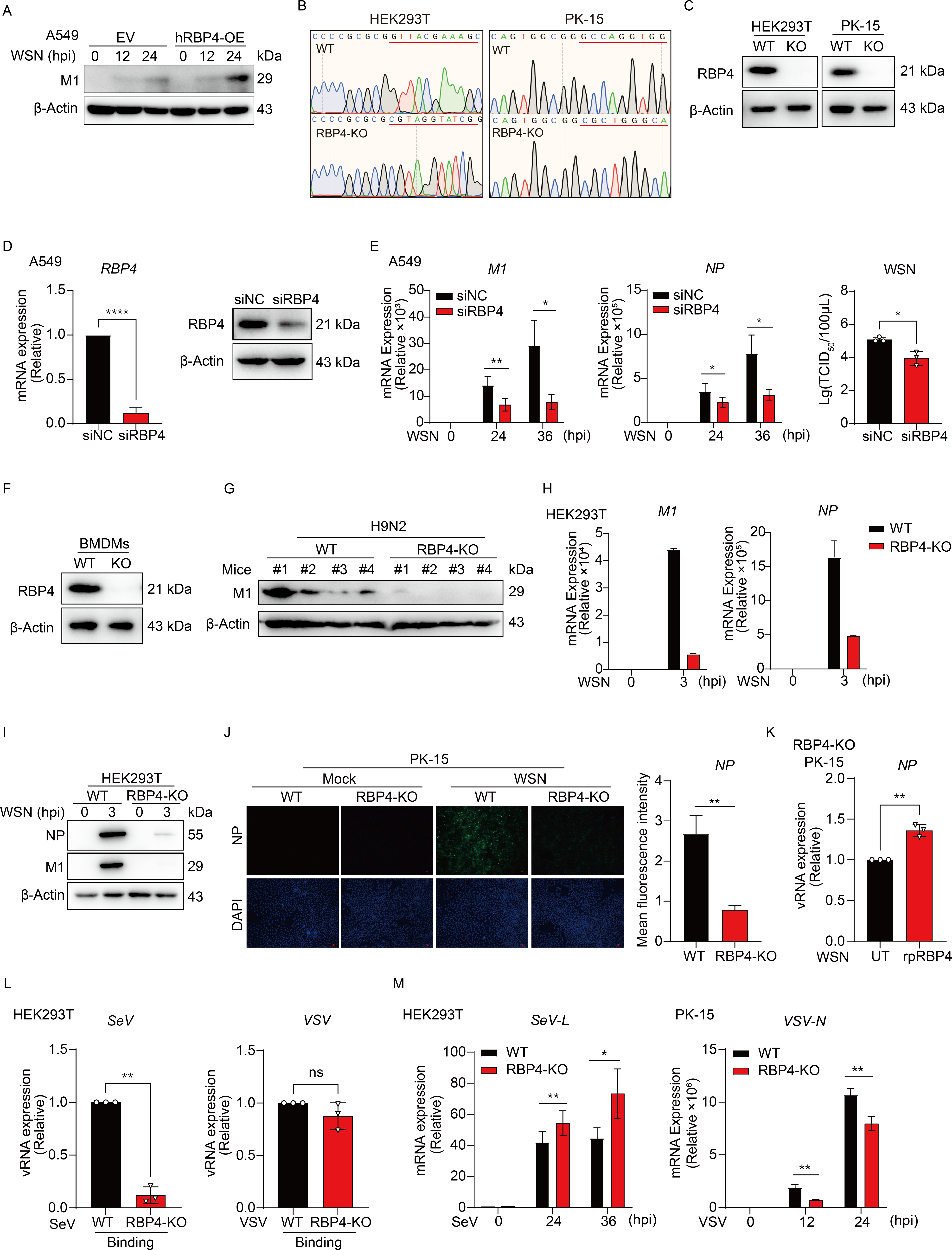

Supplement: S2 Fig — (A) Immunoblotting analysis of M1 protein levels in A549 cells transfected with RBP4 or control plasmids for 12 hours, following infection with WSN (MOI = 0.1) for indicated periods. (B, C) Sequencing validation (B) and immunoblotting analysis (C) confirming RBP4 knockout in clonal HEK293T and PK-15 cell lines. (D) qPCR or immunoblotting analysis of RBP4 mRNA or protein expression in A549 cells transfected with siRNA for RBP4. (E) qPCR analysis of mRNA levels of M1 and NP (left and middle) in A549 cells transfected as in (D) and then infected with WSN (MOI = 0.1) for the indicated periods. Viral titers (right) were determined by plaque assay at 24 hours post infection. (F) Immunoblotting analysis of RBP4 protein expression in WT and RBP4-deficient BMDMs. (G) Immunoblotting analysis of M1 protein levels in the lungs from WT and RBP4-deficient mice 3 days post-H9N2 IAV infection. Numbers indicated individual mice. (H, I) qPCR (H) or immunoblotting (I) analysis of NP and M1 mRNA and protein levels in WT and RBP4-defecient HEK293T cells infected with WSN strain (MOI = 5) for 3 hours. (J) Immunofluorescence staining of NP proteins (green) in WSN-infected (MOI = 5, 3 hours) WT and RBP4-deficient PK-15 cells. Nuclei were counterstained with DAPI (blue). Right panel shows mean fluorescence intensity from three independent experiments. Scale bars, 10 μm. (K) qPCR analysis of NP vRNA levels in RBP4-deficient PK-15 cells infected with WSN strain (MOI = 5) at the attachment stage, following treatment with recombinant porcine RBP4 (rpRBP4). (L) qPCR analysis of vRNA levels of SeV (left) or VSV (right) in WT and RBP4-deficient HEK293T cells infected with SeV (MOI = 5) or VSV (MOI = 5) for attachment assay. (M) qPCR analysis of L mRNA expression (left) in WT and RBP4-deficient HEK293T infected with SeV (MOI = 1), or N mRNA expression (right) in WT and RBP4-deficient PK-15 cells infected with VSV (MOI = 0.1) for indicated periods. Data are pooled from three independent experiments [file ppat.1013623.s002.tif]

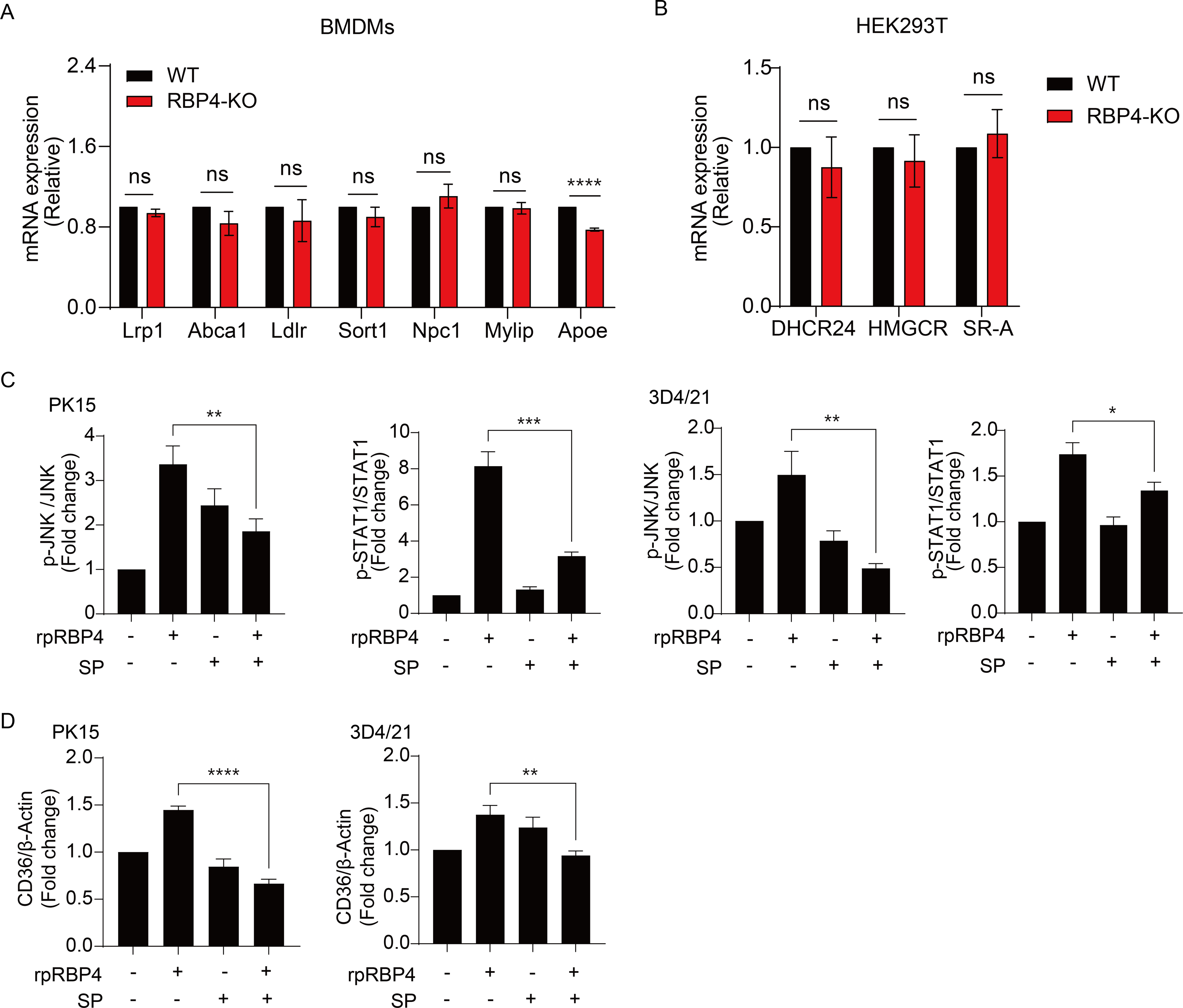

Supplement: S3 Fig — (A) qPCR analysis of selected differential genes (from the heatmap in Fig 4C) in BMDMs from WT and RBP4-deficient mice. (B) qPCR analysis of HMGCR, DHCR24 and SR-A mRNA levels in WT and RBP4-KO HEK293T cells. (C, D) Quantification of p-JNK, p-STAT1 and CD36 protein levels (normalized to total JNK, STAT1 or β-Actin, respectively) as in Fig 4H and 4I. Data were pooled from three independent experiments (A and B). *p < 0.05, **p < 0.01, ***p < 0.001, ****p < 0.0001, ns, not significant (Student’s t-test). (TIF) [file ppat.1013623.s003.tif]

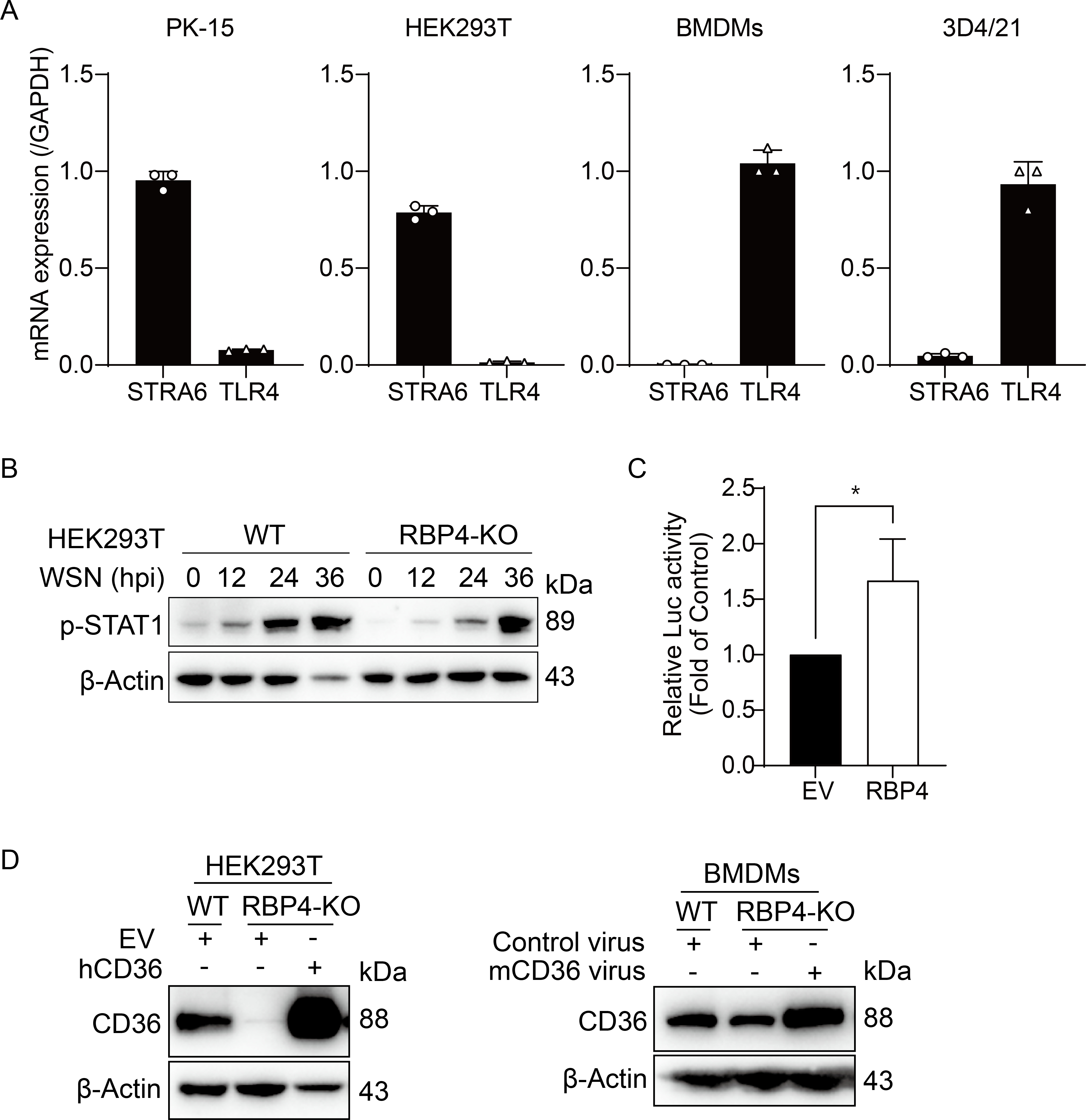

Supplement: S4 Fig — (A) qPCR analysis of STRA6 and TLR4 mRNA levels in PK-15, HEK293T, BMDMs, and 3D4/21 cells. (B) Immunoblotting analysis of p-STAT1 protein levels in WT and RBP4-deficient HEK293T cells infected with WSN (MOI = 0.1) at indicated periods. (C) Luciferase reporter assay in HEK293T cells co-transfected with hCD36 promoter-driven reporter plasmid and either an RBP4 expression plasmid or empty vector. Luciferase activity was measured 24 hours post-transfection. (D) Immunoblotting analysis of CD36 protein levels in WT, RBP4-deficient and CD36 overexpressing RBP4-deficient HEK293T cells, as well as murine CD36 retrovirus-transduced BMDMs. Data are pooled from three independent experiments (C, mean ± SD). *p < 0.05 (Student’s t-test). (TIF) [file ppat.1013623.s004.tif]
