# Supplementary material for: Retinol binding protein 4 enhances cellular cholesterol uptake to facilitate influenza A virus infection
Source: PLoS Pathog. 2025 Oct 27;21(10):e1013623. doi: 10.1371/journal.ppat.1013623 (PMC12558542; doi:10.1371/journal.ppat.1013623)
Supplement: S1 Table — (DOCX) [file ppat.1013623.s005.docx]

**S1 Table:** Primers sequences used in this study

| **Items** | **Forward sequence (5′–3′)** | **Reverse sequence (5′–3′)** |
| --- | --- | --- |
| Plasmids | Primers for plasmid construction | |
| pCMV-Myc-hCD36 | GGAATTCGGATGGGCTGTGACCGGAACT | CCGCTCGAGTTATTTTATTGTTTTCGATCT |
| pMX-puro-mCD36 | CGGGATCCATGGGCTGTGATCGGAACT | CCGCTCGAGTTATTTTCCATTCTTGGATTTGCA |
| pCDH-3×Flag-puro-mCD36 | GGAATTCATGGGCTGTGATCGGAACT | CGGGATCCTTATTTTCCATTCTTGGATTTGCA |
| pGL3-hCD36-promoter | GGTACCTTTTGGTTGAAGAAATTTAAAGAGTT | AGATCTTTCAATCAAATGCTCCAACA |
| pCDNA3.0-Flag-hRBP4 | CCCAAGCTTATGAAGTGGGTGTG | CCGCTCGAGCTACAAAAGGTTTC |
| Genes | Primers of qPCR analysis | |
| *hRBP4* | GCCTCTTTCTGCAGGACAAC | GCACACGTCCCAGTTATTCA |
| *hCD36* | ACGCTGAGGACAACACAGTC | GCCACAGCCAGATTGAGAAC |
| *hSTRA6* | GAACCTCCTTTGCAGGAAGAA | GAGTGTAGATGCAGTGTCTCAAG |
| *hTLR4* | TGAGCAGTCGTGCTGGTATC | CAGGGCTTTTCTGAGTCGTC |
| *hHMGCR* | GACGCAACCTTTATATCCGTTT | TTGAAAGTGCTTTCTCTGTACCC |
| *hDHCR24* | CTACTACCACCGCCACACG | GTTGTTGCCAAAGGGGATAA |
| *hSR-A* | TAGGCACTTGGGATGTCTGA | GTCCTCAATTTGTATTGGTGCT |
| *hGAPDH* | GAGCCACATCGCTCAGACAC | CATGTAGTTGAGGTCAATGAAG |
| *mIl6* | AGGCATAACGCACTAGGTTT | AGCTGGAGTCACAGAAGGAG |
| *mTnfa* | GCACAGAAAGCATGATCC | TAGACAGAAGAGCGTGGTGG |
| *mIl1b* | TTAGAAACAGTCCAGCCCATA | ACATCAGCACCTCACAAGCA |
| *mCd36* | GGCTGTGTTTGGAGGCATTC | TTTGCCACGTCATCTGGGTT |
| *mStra6* | GAACCTCCTTTGCAGGAAGAA | GAGTGTAGATGCAGTGTCTCAAG |
| *mTlr4* | ACAAGGCATGGCATGGCTTACAC | TGTCTCCACAGCCACCAGATTCTC |
| *mLrp1* | CTCCTGCACCATGAACAGCAAG | TAACAACCTGCTCCTCGCACC |
| *mAbca1* | GCTTGTTGGCCTCAGTTAAGG | GTAGCTCAGGCGTACAGAGAT |
| *mLdlr* | TGACTCAGACGAACAAGGCTG | CTAACTAAACACCAGACAGAGGC |
| *mSort1* | ACTTCACTGGGCTTGCTTCC | CCTCTTCACAATTCCGCTCA |
| *mNpc1* | TGTTTGGTATGGAGAGTGTGGA | GTCACAGCAGAGACTGACATTG |
| *mApoe* | CTGACAGGATGCCTAGCCG | CGCAGGTAATCCCAGAAGC |
| *mMylip* | CAGCTCCACTTTGAACAGCATC | CCACTCTATGCCGTAGTTCTCC |
| *mGAPDH* | ATCAAGAAGGTGGTGAAGCA | AGACAACCTGGTCCTCAGTGT |
| *pRBP4* | GGGCGTAGCATCCTTCCT | TCCGATTTGCCATCACAG |
| *pSTRA6* | GCAGCCACCTTCCTTCTCTT | TAGGTACGGTAGCCAGGGTC |
| *pTLR4* | TTCCGTGGCATTTTTGCTGG | ATGCCCTCTGGGATACCTGT |
| *pGAPDH* | CCTCCCCGTTCGACAGAC | TGCGGCCAAATCCGTTC |
| WSN-M1 | GACCAATCCTGTCACCTC | GATCTCCGTTCCCATTAAGAG |
| WSN-NP | TGTGTATGGACCTGCCGTAGC | CCATCCACACCAGTTGACTCTTG |
| IAV-NP-vRNA | GGCCGTCATGGTGGCGAAT | CTCAATATGAGTGCAGACCGTGCT |
| PR8-M1 | ATATACAACAGGATGGGGGCT | ATTTGCCTATGAGACCGATGCT |
| PR8-NP | GGCCGTCATGGTGGCGAAT | CTCAATATGAGTGCAGACCGTGCT |
| H3N2-NP | CCACAAGAGGGGTCCAGATT | GGAGATTTCGCTGCACTGAG |
| H9N2-NP | TGGAATCTGARGGAACTTACAAAAT | AAGGCAGCRAACCCCATTGCA |
| SeV-L | TATCAGCCCGACCGTAAGAG | AGGGTCTGGAGCCCAATCTA |
| VSV-N | TGATAGTACCGGAGGATTGACGAC | CCTTGCAGTGACATGACTGCTCTT |
